# Comparison of deep learning approaches for extreme low-SNR image restoration

**DOI:** 10.1093/gigascience/giag071

**Published:** 2026-06-22

**Authors:** Nasreen Elizabeth Buhn, Sriya Reddy Adunur, Joseph Hamilton, Summer Levis, Guy M Hagen, Jonathan D Ventura

**Affiliations:** Biological Sciences Department, California Polytechnic State University, 1 Grand Ave, San Luis Obispo, CA 93407-0401, USA; Department of Computer Science and Software Engineering, California Polytechnic State University, 1 Grand Ave, San Luis Obispo, CA 93407-0354, USA; UCCS BioFrontiers Center, University of Colorado at Colorado Springs, 1420 Austin Bluffs Parkway, Colorado Springs, CO 80918, USA; UCCS BioFrontiers Center, University of Colorado at Colorado Springs, 1420 Austin Bluffs Parkway, Colorado Springs, CO 80918, USA; UCCS BioFrontiers Center, University of Colorado at Colorado Springs, 1420 Austin Bluffs Parkway, Colorado Springs, CO 80918, USA; Department of Computer Science and Software Engineering, California Polytechnic State University, 1 Grand Ave, San Luis Obispo, CA 93407-0354, USA

**Keywords:** fluorescence microscopy, image restoration, deep learning, image stitching, phototoxicity, denoising

## Abstract

**Background:**

Live-cell fluorescence microscopy enables the study of dynamic cellular processes. However, fluorescence microscopy can damage cells and disrupt these dynamic processes through photobleaching and phototoxicity. Reducing a sample’s light exposure mitigates the effects of photobleaching and phototoxicity but results in low signal-to-noise ratio (SNR) images. Deep learning provides a solution for restoring these low-SNR images. However, these deep learning methods require large, representative datasets for training, testing, and benchmarking, as well as substantial GPU memory, particularly for denoising large images.

**Results:**

We present a new fluorescence microscopy dataset designed to expand the range of imaging conditions and specimens currently available for evaluating denoising methods. The dataset contains 17,568 paired high/low-SNR images across 15 sub-datasets that vary in specimen, imaging modality, objective, staining type, excitation wavelength, and exposure time. We evaluated five state-of-the-art deep learning denoising models on the dataset, including supervised, unsupervised, and zero-shot techniques. We also developed an image stitching method that enables large images to be processed in smaller crops and reconstructed.

**Conclusions:**

Our dataset provides a diverse benchmark for evaluating deep learning denoising methods, and our stitching method provides a solution to GPU memory constraints encountered when processing large images. Among the evaluated deep learning models, the supervised Transformer-based model had the best denoising performance but required the longest training time.

## Background

The imaging of live cells and tissues is an essential process that enables scientists to observe dynamic cellular activity. Live imaging is commonly performed using fluorescence microscopy, which allows for the detection and tracking of biological molecules with high sensitivity and specificity [1]. These biological molecules are probed with fluorophores, which are excited by distinct wavelengths of light to produce emissions used to generate images. However, during the excitation of fluorophores, photobleaching and phototoxicity can occur, which introduce challenges to the consistency and reproducibility of imaging data [2].

The excitation of fluorophores can result in damage to their chemical structure, a process known as photobleaching. As fluorophores undergo photobleaching, they may interact with oxygen, generating reactive oxygen species (ROS) [2]. An unnatural increase in ROS can cause phototoxicity, leading to detrimental changes in a specimen, including damage to DNA, induced mutations, oxidized proteins, and potential disruption of the developmental processes within a cell [2].

To produce reliable data, phototoxicity must be minimized. Accordingly, numerous approaches have been developed to mitigate its effects. Many strategies focus on reducing a sample’s light exposure by modifying microscope hardware, sample environment, and imaging conditions. One approach has been limiting light exposure to areas outside the focal plane. This approach serves as the basis for multiple fluorescence microscopy modalities, including total internal reflection fluorescence, lateral sheet fluorescence microscopy, and two-photon microscopy [3]. Despite these methods, phototoxicity remains a challenge, since high local light intensities can still damage cells, especially during prolonged live imaging [2].

Reducing excitation light intensity and/or exposure times can minimize photobleaching and phototoxicity but leads to low signal-to-noise ratio (SNR) images. To circumvent this, computational image restoration techniques have been applied to restore low SNR images. However, traditional restoration algorithms, such as BM3D, which rely on predefined mathematical models and heuristics, struggle to address complex noise patterns [4]. In contrast, deep learning models can learn the structure of complex noise types, including cases with unknown noise levels [5]. Recent work by Hagen et al. demonstrates that these deep learning models outperform traditional models in terms of quantitative metrics and visual quality [6].

Deep learning is a subset of machine learning that uses artificial neural networks, computational networks inspired by the brain, to learn patterns from large datasets. These networks rely on layers of interconnected nodes to transform input data into outputs using learned weights. Among deep learning methods, convolutional neural networks (CNNs) [7] have been proven effective for image-related tasks. CNNs use filters to extract image features like edges, textures, and shapes, allowing the network to learn visual patterns. More recently, Transformer-based models, initially developed for natural language processing [8], have been adapted for vision tasks [9], offering advantages in long-range dependencies and global contexts compared to CNNs.

Deep learning approaches for image restoration can be classified into three major categories: supervised, self-supervised, and zero-shot [10]. Supervised methods rely on large datasets of paired low and high SNR images to learn the relationship between noisy and clean images. In contrast, self-supervised methods do not rely on ground truth clean images, and instead learn denoising from noisy data alone. Zero-shot methods denoise a single noisy image without training on a representative dataset. Extensive evaluation datasets are required to benchmark the performance of supervised, self-supervised, and zero-shot methods.

To meet the data requirements necessary for denoising models, datasets should span fluorescence microscopy imaging modalities and biological specimens [6, 11–14]. Such datasets should capture complex noise patterns arising from varying imaging conditions and specimens, enabling supervised models to learn robust denoising mappings. This range of conditions allows for the assessment of model generalizability and helps prevent overfitting to specific training data. These datasets serve as valuable benchmarks for comparing deep learning methods for fluorescence microscopy image denoising.

While dataset diversity is important for training and evaluating fluorescence microscopy denoising methods, few datasets are currently available. The FMD dataset, introduced by Zhang et al. [11], consists of 12,000 images spanning fluorescence microscopy modalities, including confocal, two-photon, and widefield. This dataset includes samples from cells, zebrafish, and mouse brain tissue, but is limited in both sample diversity and image quality. The W2S dataset introduced by Zhou et al. [12] provides 360 image sets with varying noise levels but is constrained to solely widefield imaging. Weigert et al. [15] provided a collection of paired image patches for two specimens to evaluate denoising methods. More recently, Hagen et al. [6] introduced a dataset consisting of 567 paired images ranging from 0.26 to 4.19 megapixels, covering both widefield and confocal imaging modalities. This dataset includes images of actin, mitochondria, nucleus, and membrane samples. However, it remains limited in terms of noise level variation, image sizes, and sample types. A well-suited dataset for training deep learning models should include a diverse range of fluorescence microscopy modalities, objective lenses, noise levels, exposure times, and biological samples.

We introduce a novel collection of 324 full-size, paired images, separated into 17,568 paired image crops, containing a greater diversity of specimens and more extreme noise levels than existing fluorescence microscopy denoising datasets. Previously unrepresented specimens include breast cancer tissue array spots, earthworm, freshwater fish gills, rat testis, rabbit testis, human ovary, and tubulin. Imaging modalities include widefield and spinning disk confocal microscopy. We include more extreme noise levels than in previous datasets by widening the gap between high and low exposure times. This increased diversity of specimens and noise conditions makes our dataset a more comprehensive and well-suited resource for evaluating the performance of fluorescence microscopy denoising models. More extreme noise poses a challenge for denoising methods, since the larger variance in the data makes it more difficult for such methods to determine the underlying clean signal from limited samples. Different specimen types pose a challenge due to the diversity of visual patterns and varying level of self-similarity in the samples.

In addition to the necessity of large, diverse datasets, deep learning approaches require substantial memory for computation. As a result, denoising of fluorescence microscopy images is limited by GPU memory constraints. To address this challenge, we introduce an image stitching approach that enables large images to be denoised in smaller crops and reassembled [16–19]. Inspired by panorama stitching techniques [20, 21], we optimize per-tile brightness and contrast terms to compensate for the output variation introduced by learning-based denoising methods, and apply linear blending in the overlap region.

We evaluated the performance of five state-of-the-art deep learning denoising models using our dataset. Our selection included supervised [15, 22], unsupervised [23, 24], and zero-shot [25] methods. BM3D [4] was excluded from our analysis due to its inferior performance compared to CARE [15] in a previous study [6]. Self-Supervised Poisson Gaussian Denoising (SSPG) [23] is an extension of blindspot denoising techniques [26, 27] to support the Poisson–Gaussian noise model, which is commonly applied in fluorescence microscopy. Self-Inspired Noise2Noise (SN2N) [24] employs an interpolation technique to generate data for Noise2Noise [28] training. SSPG and SN2N are self-supervised techniques to learn a denoiser from noisy data alone. Noise2Fast, proposed by Lequyer et al. [25], is a zero-shot unsupervised denoising method, meaning that it does not require any training on a separate dataset, and instead learns to denoise directly from a single input image. Noise2Fast relies on a checkerboard-down sampling technique to generate a set of four images from the input noisy image, which are used to train a lightweight feed-forward neural network using Noise2Noise-style self-supervision [28]. Content Aware Image Restoration (CARE), developed by Weigert et al. [15], is a supervised model that utilizes a U-Net architecture [29, 30] to learn mappings from degraded images to denoised versions. Restormer, introduced by Zamir et al. [22], is a supervised denoising model that employs an encoder-decoder Transformer-based architecture. The model’s core components include a multi-Dconv head transposed attention (MDTA) block and a gated Dconv feed-forward network (GDFN). MDTA aids the model in learning both fine details and broader patterns by combining attention mechanisms and depth-wise convolutions. GDFN improves feature quality by using gates and convolutional layers to refine and enhance each layer’s image content. Transformer-based models have outperformed the more commonly used U-Net in previous studies [31].

## Data description

We generated fifteen distinct datasets of paired low- and high-SNR images composed of specimens from actin, breast cancer array, earthworm, fish gill, rat testes, immature ovaries, human ovaries in active phase, mitochondria, mouse brain, rabbit testes, and tubulin as detailed in Table 1. There are 324 images in total, ranging in size from 4.19 to 282.22 megapixels (MP). Imaging conditions varied in light intensity, exposure times, objectives, excitation wavelengths, and stains as detailed in Table 2. We extracted non-overlapping crops of size $512\times 512$ pixels from the images, resulting in a total of 17,568 crops, which we then divided into a 90%/10% train/test split for each dataset.

**Table 1.** Overview of the samples and preparations in our dataset.

Dataset(s)	Sample type	Stain	Sample source	Catalog number
01	Actin	Alexa488-pahlloidin	Invitrogen	Fluoroslides #1
02	Breast Cancer Array	H&E Stain	Tissuearray.com	BR249
03	Earthworm	H&E Stain	Eisco	BS18225
$04-06$	Fish Gill	H&E Stain	Eisco	BS18101
$07-08$	Rat Testis	H&E Stain	Carolina	316464
$09-10$	Human Ovary	H&E Stain	Carolina	616024
11	Immature Ovary	H&E Stain	Eisco	BS18222
12	Mitochondria	MitoTracker Red	Invitrogen	Fluoroslides #1
13	Mouse Brain	GFP	Sunjin Lab	-
14	Rabbit Testis	H&E Stain	Amscope	PS50
15	Tubulin	BODIPY FL-GAM	Invitrogen	Fluoroslides #2

**Table 2.** Overview of dataset imaging conditions.

		No.		No.	Exp. Time					
Dataset	Type	Images	Sizes [MP]	Crops	(Low/High) [ms]	Obj. Mag./NA	Ex. [nm]	Em. [nm]	*a*	*b*
01	WF	23	5–54.86	1,552	1/100	60$\times$/1.42	470	525	0.0092	0.0003
02	WF	1	282	1,024	1/100	60$\times$/1.42	530	575	0.0091	$-$ 0.0007
03	WF	1	134	400	1/600	20$\times$/0.45	530	575	0.0321	$-$ 0.0116
04	WF	1	256	576	1/100	20$\times$/0.45	530	575	0.0116	$-$ 0.0009
05	WF	12	19–68	1,328	1/300	10$\times$/0.40	530	575	0.0419	$-$ 0.0152
06	WF	11	28–55	1,344	1/600	10$\times$/0.40	530	575	0.0385	$-$ 0.0148
07	CF	105	4	1,680	20/200	60$\times$/1.35	532	575	0.0023	$-$ 0.0001
08	CF	100	4	1,600	20/500	60 $\times$/1.35	450	575	0.0146	$-$ 0.0032
09	WF	13	28–72	1,424	1/100	10$\times$/0.4	530	575	0.0171	$-$ 0.0028
10	WF	11	28–55	1,344	1/100	4$\times$/0.16	530	575	0.0177	$-$ 0.0025
11	WF	1	90	256	1/600	20$\times$/0.45	530	575	0.0356	$-$ 0.0123
12	WF	14	5–55	1,568	1/100	60$\times$/1.42	530	575	0.0197	$-$ 0.0048
13	WF	8	36–126	1,216	1/100	10$\times$/0.4	470	515	0.0327	$-$ 0.0095
14	WF	9	5–81	928	1/100	10$\times$/0.40	530	575	0.0137	$-$ 0.0015
15	WF	14	5–55	1,328	1/100	60$\times$/1.42	470	515	0.0215	$-$ 0.0045

WF indicates widefield and CF indicates spinning disk confocal.

We estimated the noise level of the images using the single-image Poisson-Gaussian noise estimation method of Foi et al. [32]. This approach models the variance of the noisy pixel value *z* in terms of the clean value *x* as $\textrm {Var}[z]=ax+b$, where *a* and *b* are the parameters of the model. We estimated the noise parameters for each image crop and report the average values for each dataset in Table 2. Compared to the fluorescence microscopy dataset (FMD) [11], our dataset has similar levels of Poisson noise (*a*) but generally an order of magnitude higher Gaussian noise (*b*).

We also estimated the noise level of the images by calculating the PSNR of the raw, low-SNR (noisy) image compared to the high-SNR (clean) image (see Table 3). The average raw PSNR of our datasets ranges from 14.12 to 27.66 dB, with an average of 19.92 dB. For comparison, the raw PSNR in the dataset of Hagen et al. [6] ranged from 18.34 to 29.4 dB, with an average of 24.34 dB, and the FMD dataset [11] had an average raw PSNR of 27.22 dB. Thus our dataset consists of more extreme low-SNR data and tests a previously unexplored boundary of what current denoising methods can handle.

**Table 3.** Average test set results.

	PSNR						SSIM					
Dataset	Noisy	SSPG	SN2N	Noise2Fast	CARE	Restormer	Noisy	SSPG	SN2N	Noise2Fast	CARE	Restormer
01	23.22	29.05	28.59	29.47	29.50	**31.01**	0.37	0.63	0.63	0.65	0.65	**0.68**
02	23.31	29.83	29.64	30.15	28.07	**34.26**	0.40	0.77	0.77	0.78	0.74	**0.82**
03	17.15	17.68	18.59	18.62	18.54	**19.89**	0.08	0.10	0.13	0.15	0.12	**0.18**
04	22.32	32.17	30.55	32.38	32.04	**33.41**	0.39	0.84	0.80	0.85	0.85	**0.87**
05	15.70	18.51	20.02	20.02	20.83	**23.90**	0.05	0.13	0.29	0.23	0.28	**0.43**
06	15.40	17.80	19.42	19.14	20.33	**23.16**	0.05	0.12	0.27	0.20	0.28	**0.42**
07	27.66	29.51	29.61	29.37	30.28	**33.73**	0.75	0.88	0.88	0.88	0.89	**0.93**
08	18.09	24.52	26.05	26.60	26.60	**27.64**	0.14	0.43	0.47	0.49	0.49	**0.53**
09	19.78	24.80	23.63	25.94	25.88	**26.80**	0.34	0.61	0.49	0.65	0.64	**0.68**
10	21.14	26.25	24.33	26.83	27.03	**28.12**	0.43	0.64	0.55	0.66	0.68	**0.72**
11	14.12	16.21	17.33	17.41	18.06	**21.43**	0.03	0.08	0.22	0.16	0.22	**0.39**
12	22.86	29.42	29.20	30.44	30.38	**32.16**	0.30	0.63	0.63	0.69	0.70	**0.75**
13	18.35	24.01	26.08	25.71	24.43	**28.24**	0.11	0.35	0.46	0.42	0.37	**0.50**
14	21.21	29.07	27.82	29.24	29.35	**30.60**	0.39	0.75	0.71	0.76	0.77	**0.79**
15	18.56	25.99	26.12	27.07	27.15	**28.73**	0.16	0.51	0.53	0.57	0.60	**0.66**

The highest values per dataset are in bold.

## Analyses

### Denoising performance

**Figure 1:**
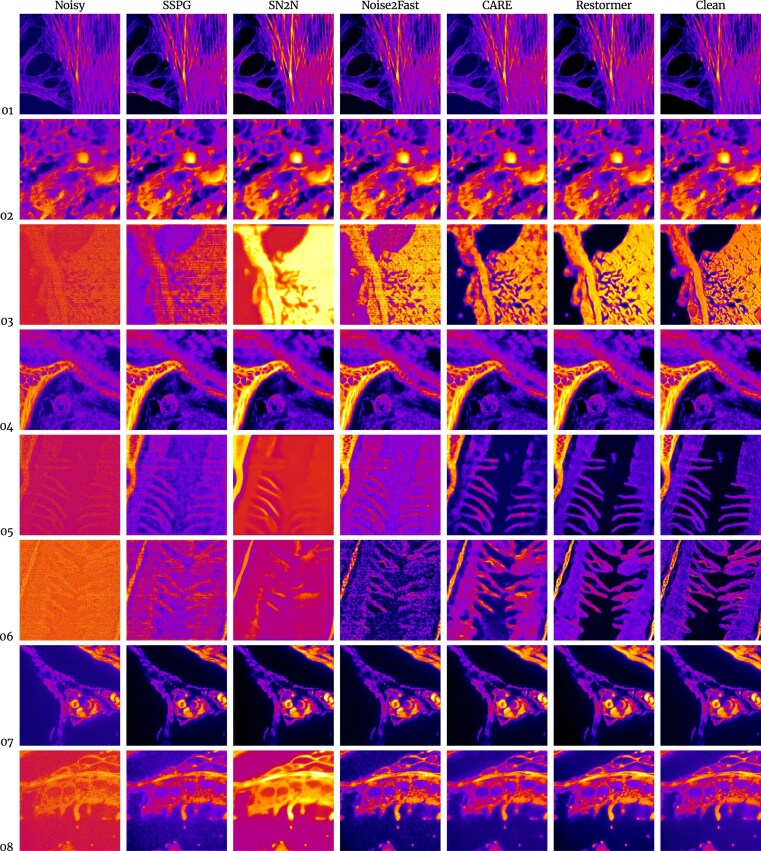
Example noisy input, denoised, and clean images.

**Figure 2:**
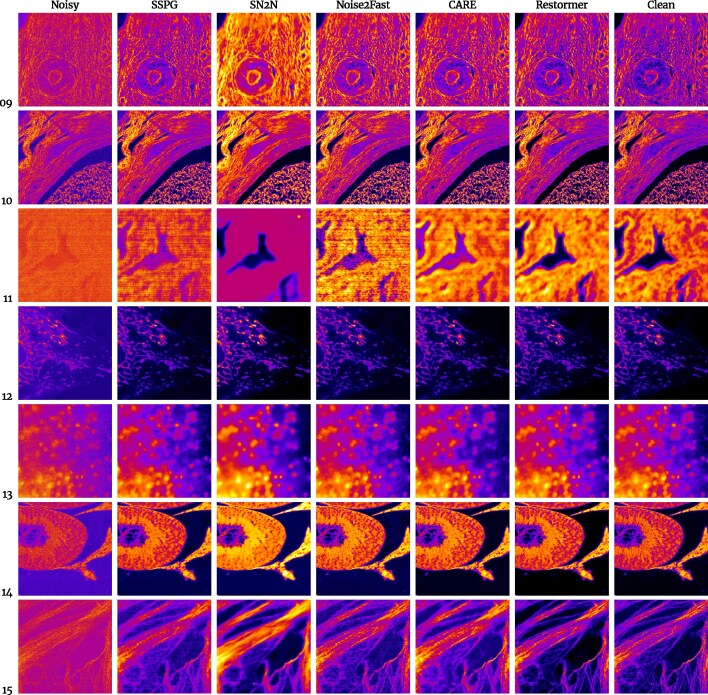
Example noisy input, denoised, and clean images.

**Figure 3:**
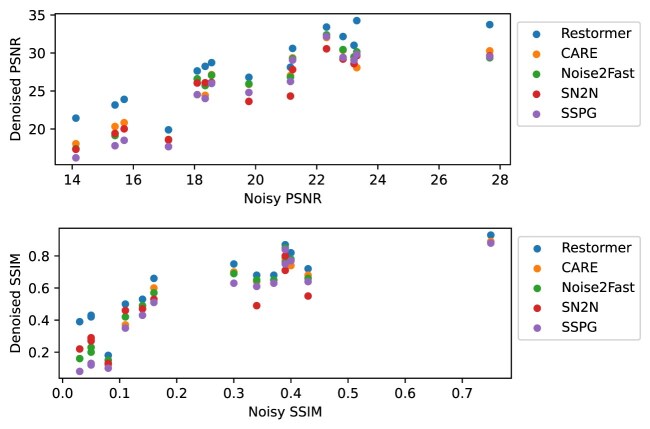
Average noisy vs. denoised PSNR and SSIM for each model and dataset.

Figs. 1 and 2 shows representative low-SNR input images, denoised outputs from each model, and the corresponding high-SNR ground truth images from each dataset, with the “fire” colormap applied to enhance contrast. The denoising performance of each method is summarized in Table 3 and visualized in Fig. 3. Restormer consistently achieved the highest average PSNR and SSIM. CARE and Noise2Fast’s comparative performance varied depending on the dataset. The performance of the unsupervised techniques SSPG and SN2N was typically behind the other methods.

### Speed

Restormer’s training times ranged from 16 to 18 hours to several days, while the CARE network trained in approximately 3.5 hours per dataset, SSPG about 1 hour per dataset, and SN2N about 8.5 hours per dataset. Noise2Fast does not require training on a separate training set. Inference times also varied between models: Restormer required about 0.45 seconds per image, CARE about 1 second per image, SSPG about 0.20 seconds per image, SN2N about 0.08 seconds per image, and Noise2Fast, due to its iterative self-supervised denoising method, between 9 and 40 seconds per image.

### Adaptive stitching

**Figure 4:**
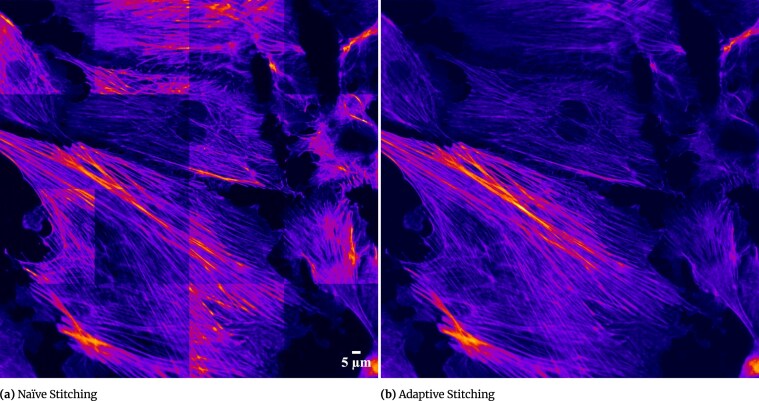
Effect of stitching logic on Restormer-denoised images from Dataset 1. (a) Naïve stitching of non-overlapping crops. (b) Adaptive stitching of overlapping crops. Final images are 2048$\times$2048 pixels.

**Figure 5:**
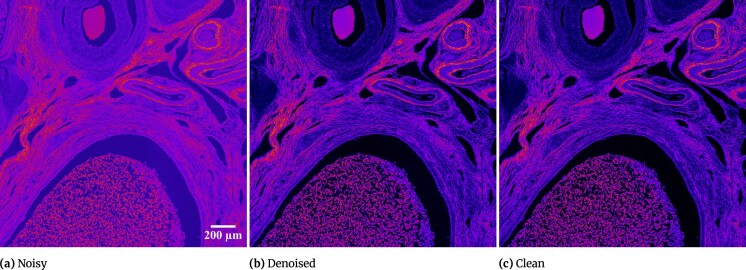
Example adaptively stitched image from Dataset 9. (a) Noisy image. (b) Adaptive stitching of Restormer denoising results. (c) Clean image. All images are 3072$\times$3072 pixels.

The deep learning models introduced variations in light intensity between neighboring denoised crops, as shown in Fig. 4. Our adaptive image stitching method reduces these intensity differences across stitched crops compared to naïve stitching. Fig. 5 shows an example adaptively stitched Restormer result compared to the corresponding low- and high-SNR images.

## Discussion

Across the specimens, imaging modalities, and noise conditions present in our dataset, Restormer achieved the highest performance in terms of PSNR and SSIM. However, due to its supervised training and Transformer-based encoder-decoder architecture, Restormer required substantially longer training times than other supervised models.

We found the denoising performance of SSPG and SN2N to be behind the other methods, which is reasonable considering that the supervised methods (CARE and Restormer) have access to clean data during training, and the zero-shot method (Noise2Fast) trains directly on the test image. Additionally, SSPG, SN2N, and Noise2Fast all make the assumption of independent and identically distributed (i.i.d.) noise, whereas our dataset contains structured noise. Thus the unsupervised and zero-shot methods we tested are likely hindered by the i.i.d. noise assumption.

While its computational costs were considerably higher, Restormer was the most effective model for removing noise while preserving the structural integrity of the original images. Preserving the fine structural detail of fluorescence microscopy images is particularly important because the introduction of artifacts compromises the reliability of downstream biological analysis. This highlights the critical trade-off between computational cost and model accuracy in fluorescence microscopy denoising.

In addition to computational requirements, supervised deep learning models rely on representative, paired image datasets to learn robust mappings from low-SNR to high-SNR images. Denoising performance depends on a model’s ability to learn these mappings, but with limited or unrepresentative training datasets, models risk overfitting to specific imaging conditions and fail to handle the inherent variability of live fluorescence microscopy imaging. In contrast, unsupervised approaches do not require paired training data, but they demonstrated inferior denoising performance, as they struggled to remove noise and maintain the structural integrity of the images in our dataset.

The deep learning models introduced variations in light intensity between the individually denoised neighboring crops, resulting in visible seams and uneven lighting in the reconstructed images. To address the issue of uneven lighting in denoised crops, we implemented an adaptive stitching approach that adjusts each image segment’s light intensity based on its neighbors. This method was able to effectively decrease intensity variation across images. Our adaptive stitching process enabled the denoising of large images using deep learning models without exceeding our memory constraints.

## Potential implications

Our results highlight the relationship between computational costs and denoising accuracy, underscoring the need for deep learning denoising models that are both computationally efficient and able to remove noise without compromising image integrity. Additionally, given the diversity of biological structures included in our dataset, it would be valuable to explore whether certain deep learning techniques are more effective for denoising specific biological structures. Lastly, since tiny structural distortions can have a significant impact on biological interpretations but may not strongly affect PSNR and SSIM, there is a need for image quality metrics specifically tailored to microscopy images [33].

## Methods

### Microscopy

We acquired the fluorescence images using an Olympus BX53 microscope equipped with a motorized XY stage (Applied Scientific Imaging, Eugene, OR), Aura III light source (Lumencor, Beaverton, OR), Fluorescence filters (Chroma, Bellows Falls, VT), and Fusion BT camera (Hamamatsu Photonics, Hamamatsu, Japan). The spinning disk confocal setup is described in our previous work [34].

### Deep learning methods

Each model was trained and tested using an Nvidia V100 32GB GPU. For each method, we used the authors’ provided implementations and default settings for training parameters such as loss function, batch size, and learning rate schedule.

For fair comparison, we used consistent data pre-processing and data augmentation procedures across all methods to the extent possible. For pre-processing during training, the images were normalized using percentile normalization. For data augmentation, we horizontally and vertically flipped the images and rotated by 90, 180, and 270 degrees. SSPG, SN2N, CARE, and Restormer were trained on patches randomly sampled from the crops. For SSPG, SN2N, and CARE, we used $128\times 128$ pixel patches. The recommended approach for Restormer is to increase patch size during training; we began with $128\times 128$ pixel patches and increased to $384\times 384$ pixels by the end of training. Noise2Fast has its own training procedure specific to its efficient and zero-shot design, and so does not use patching or data augmentation.

### Image quality metrics

We analyzed the performance of the models on a holdout test set from each dataset not used during training. Performance was quantified using peak signal-to-noise ratio (PSNR) and structural similarity index measure (SSIM) [35] by comparing each denoised result to its corresponding ground truth image. PSNR measures the ratio of the maximum signal power in an image to the power of the noise present in the image. SSIM is based on human visual perception of an image and evaluates image similarity using contrast, luminance, and structure. SSIM values range from zero to one. Higher PSNR and SSIM values indicate increased image similarity and, thus, greater denoising success.

Prior to calculating PSNR and SSIM, images were normalized using the “minimum MSE” normalization method ([15], Supplementary Notes, Section 2.2).

### Adaptive image stitching

**Figure 6:**
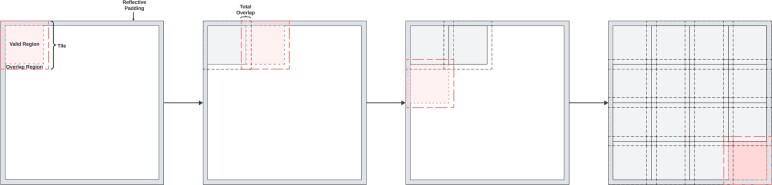
Image cropping logic. The highlighted tile shows the progression of the cropping logic. The shaded area marks the valid region of each tile, while the overlapping region is the space between the valid region and the outer dashed line. Cropping logic proceeds until the entire image is processed.

We prepared the full-size test images for stitching by adding 64-pixel reflective padding around the borders and then dividing the images into $640\times 640$ pixel overlapping crops, so that each crop overlapped with its neighbors by 64 pixels, as illustrated in Fig. 6. The central region of each crop, excluding the overlap, represents the valid region of $512\times 512$ pixels.

Crops were denoised using the trained Restormer model, then processed in our adaptive stitching algorithm to re-assemble the crops back into a full-size composite denoised image. We optimized the agreement between overlapping regions by adjusting each crop’s scale and shift using the trust region reflective algorithm over a least squares objective. To anchor the adaptive stitching algorithm, we identified the noisy crop with the greatest intensity and assigned its corresponding crop in the denoised image a fixed scale and shift. Following intensity adjustment, a feathered weight mask was applied to each crop, with the weight linearly decreasing from the center of each crop to its edges. In overlapping regions, pixels from all neighboring crops were summed and divided by the total weight at each position, producing smooth transitions between crops.

## Supplementary Material

giag071_Authors_Response_To_Reviewer_Comments_original_submission

giag071_Authors_Response_To_Reviewer_Comments_revision_1

giag071_GIGA-D-25-00430_original_submission

giag071_GIGA-D-25-00430_revision_1

giag071_GIGA-D-25-00430_revision_2

giag071_Reviewer_1_Report_original_submission Reviewer 1 -- 1/4/2026

giag071_Reviewer_1_Report_revision_1 Reviewer 1 -- 4/20/2026

giag071_Reviewer_2_Report_original_submission Reviewer 2 -- 1/13/2026

## Data Availability

All raw and analyzed data [36] is available on GigaDB [37]. All files and data are distributed under the Creative Commons CC0 waiver, with a request for attribution. DOME-ML (Data, Optimisation, Model, and Evaluation in Machine Learning) annotation supporting the current study is available through DOME Registry [38].

## Availability of source code and requirements

- Project name: Comparison of Deep Learning Approaches for Extreme Low-SNR Image Restoration software and analytics code
- Project home page: https://github.com/nazbuhn/extreme-low-snr-restoration
- Operating systems(s): Platform independent
- Programming language(s): Python
- License: MIT License

## Abbreviations

BM3D: block-matching and 3D filtering; CARE: content-aware image restoration; CNN: convolutional neural network; GDFN: Gated-Dcov Feed-Forward Network; GPU: graphics processing unit; MDTA: multi-Dconv transposed attention; MP: megapixels; NA: numerical aperture; PSNR: peak signal-to-noise ratio; ROS: reactive oxygen species; SN2N: Self-Inspired Noise2Noise; SNR: signal-to-noise ratio; SSIM: structural similarity index measure; SSPG: Self-Supervised Poisson-Gaussian Denoising.
