# Supplementary material for: Comparison of deep learning approaches for extreme low-SNR image restoration
Source: Gigascience. 2026 Jun 22;15:giag071. doi: 10.1093/gigascience/giag071 (PMC13366542; doi:10.1093/gigascience/giag071)
Supplement: giag071_GIGA-D-25-00430_revision_2 [file giag071_giga-d-25-00430_revision_2.pdf]

GigaScience

Comparison of Deep Learning Approaches for Extreme Low-SNR Image Restoration
--Manuscript Draft--

**Manuscript Number:**	GIGA-D-25-00430R2
**Full Title:**	Comparison of Deep Learning Approaches for Extreme Low-SNR Image Restoration
**Article Type:**	Research
**Funding Information:**	National Institute of General Medical Sciences (2R15GM128166-02) — Dr. Guy M. Hagen
**Abstract:**	Background: Live-cell fluorescence microscopy enables the study of dynamic cellular processes. However, fluorescence microscopy can damage cells and disrupt these dynamic processes through photobleaching and phototoxicity. Reducing a sample's light exposure mitigates the effects of photobleaching and phototoxicity but results in low signal-to-noise ratio (SNR) images. Deep learning provides a solution for restoring these low-SNR images. However, these deep learning methods require large, representative datasets for training, testing, and benchmarking, as well as substantial GPU memory, particularly for denoising large images. Results: We present a new fluorescence microscopy dataset designed to expand the range of imaging conditions and specimens currently available for evaluating denoising methods. The dataset contains 17,568 paired high/low-SNR images across 15 sub-datasets that vary in specimen, imaging modality, objective, staining type, excitation wavelength, and exposure time. We evaluated six state-of-the-art deep learning denoising models on the dataset, including supervised, unsupervised, and zero-shot techniques. We also developed an image stitching method that enables large images to be processed in smaller crops and reconstructed. Conclusions: Our dataset provides a diverse benchmark for evaluating deep learning denoising methods, and our stitching method provides a solution to GPU memory constraints encountered when processing large images. Among the evaluated deep learning models, the supervised Transformer-based model had the best denoising performance but required the longest training time.
**Corresponding Author:**	Nasreen Buhn<br>California Polytechnic State University<br>San Luis Obispo, CA UNITED STATES
**Corresponding Author Secondary Information:**	
**Corresponding Author's Institution:**	California Polytechnic State University
**Corresponding Author's Secondary Institution:**	
**First Author:**	Nasreen Buhn
**First Author Secondary Information:**	
**Order of Authors:**	Nasreen Buhn
	Sriya Reddy Adunur
	Joseph Hamilton
	Summer Levis
	Guy M. Hagen
	Jonathan D. Ventura
**Order of Authors Secondary Information:**	
**Response to Reviewers:**	Response to Reviewers<br><br>We would like to again thank the reviewers for their careful consideration of our manuscript and thoughtful suggestions for improvement. We have revised our

	manuscript to address reviewers' comments and provided a version of the revised manuscript with changes marked in blue. A detailed response to each review comment is provided below.<br><br>Reviewer #1<br><br>"The authors state that Self-inspired Noise2Noise (SN2N) was tested on two datasets, but "performance was far behind all other methods," and therefore excluded it from the benchmark. However, this conclusion provides no quantitative metrics such as PSNR/SSIM; no training iterations, learning rate, or other key hyperparameter configurations and model convergence information; and no clarification on whether the official implementation or a custom reimplementation was used. N2N methods are unstable and sensitive to hyperparameters. Without the above details, it is impossible to rule out the possibility that performance was underestimated due to insufficient training or improper implementation. It is suggested that the authors clarify the code source and re-evaluate SN2N's performance."<br><br>Thank you for these comments. We apologize for the lack of details in our previous response.<br><br>We used the authors' implementation of SN2N provided at https://github.com/SR-Wiki/SN2N without modification, following their suggested default settings. To align with the recommendations of SN2N and the preprocessing used for the other methods, we first applied percentile normalization to the images using the default parameters suggested by SN2N. To generate the corresponding patches for N2N training, we used their 2D patch generation script, including eight-fold dataset augmentation with rotations and flips, as used for the other methods tested. We did not apply Patch2Patch data augmentation, following the default settings specified by SN2N and for consistency with the other methods tested. Training was performed according to the defaults specified by SN2N: self-constrained loss weight of 1, batch size of 32, learning rate of 2e-4, and 100 epochs.<br><br>We trained and tested SN2N on all our datasets. We updated Table 3 with the SN2N results and Figures 1 and 2 with example images. In general, SN2N was either the worst or second-to-worst performer.<br><br>In our discussion section we noted that SSPG, SN2N, and Noise2Fast all make the assumption of independent and identically distributed (i.i.d.) noise, whereas our dataset contains structured noise. Thus the unsupervised methods we tested are likely hindered by the i.i.d. noise assumption.<br><br>"In response to the reviewer's request for characterization of underlying noise statistics, the authors replied using PSNR to estimate noise levels. However, PSNR is an image quality assessment metric that measures the difference between noisy and clean images, rather than the statistical distribution properties of the noise itself (such as Poisson-Gaussian parameters). It is suggested that the authors supplement the variance-mean analysis to estimate Poisson-Gaussian parameters."<br><br>Thank you for this suggestion. Following the methods of Zhang et al. (2019) we chose to estimate the Poisson-Gaussian noise model parameters using the single-image estimation method of Foi et al. (2008). We report the average an and b parameter estimates in Table 2 and added a discussion of the results to the Data Description section. Compared to the Fluorescence Microscopy Dataset (Zhang et al., 2019), our dataset has similar levels of Poisson noise (a) but generally an order of magnitude higher Gaussian noise (b).
**Additional Information:**	
**Question**	**Response**
Are you submitting this manuscript to a special series or article collection?	No
**Experimental design and statistics**	Yes

Powered by Editorial Manager® and ProduXion Manager® from Aries Systems Corporation

Full details of the experimental design and statistical methods used should be given in the Methods section, as detailed in our Minimum Standards Reporting Checklist. Information essential to interpreting the data presented should be made available in the figure legends.<br><br>Have you included all the information requested in your manuscript?	
**Resources**<br><br>A description of all resources used, including antibodies, cell lines, animals and software tools, with enough information to allow them to be uniquely identified, should be included in the Methods section. Authors are strongly encouraged to cite Research Resource Identifiers (RRIDs) for antibodies, model organisms and tools, where possible.<br><br>Have you included the information requested as detailed in our Minimum Standards Reporting Checklist?	Yes
**Availability of data and materials**<br><br>All datasets and code on which the conclusions of the paper rely must be either included in your submission or deposited in publicly available repositories (where available and ethically appropriate), referencing such data using a unique identifier in the references and in the “Availability of Data and Materials” section of your manuscript.<br><br>Have you have met the above requirement as detailed in our Minimum Standards Reporting Checklist?	Yes
GigaScience has policies and guidelines in place for the use of generative AI-	No

Powered by Editorial Manager® and ProduXion Manager® from Aries Systems Corporation

writing tools such as ChatGPT. If you have used such writing tools to assist with writing the manuscript this must be declared and cited in the text. Authors should not list AI-writing tools and other AI-assisted technologies as an author or co-author and should acknowledge that they are fully responsible for text generated or refined by AI-writing tools.<p><br><br>A summary of use (particularly in the introduction or among methods) needs to be included at the end of the paper, and the outputs should also be included as a supplementary file hosted in GigaDB or other open repositories. Please read our guidelines for more information. <p><br><br>By submitting to GigaScience, you are aware of the journal's AI-writing tools policy, and if you have declared use of such tools below, you have acknowledged this where appropriate in your manuscript and have made a summary of use and outputs available. <p><br><b>AI-assisted writing tools have been used in the preparation of this manuscript?	

Manuscript

Click here to access/download;Manuscript;Gigascience_2026 (1).pdf

Placeholder for OUP logo oup.pdf

GigaScience, 2023, 1–9

doi: xx.xxxx/xxxx
Manuscript in Preparation
Research

RESEARCH

Comparison of Deep Learning Approaches for Extreme Low-SNR Image Restoration

Nasreen Elizabeth Buhn[1,*], Sriya Reddy Adunur[2,†], Joseph Hamilton[3,†], Summer Levis[3,†], Guy M. Hagen[3,†] and Jonathan D. Ventura[2,†]

[1]Biological Sciences Department, California Polytechnic State University, San Luis Obispo, California, 93407 and [2]Department of Computer Science and Software Engineering, California Polytechnic State University, San Luis Obispo, California, 93407 and [3]UCCS BioFrontiers Center, University of Colorado at Colorado Springs, 1420 Austin Bluffs Parkway, Colorado Springs, Colorado, 80918

*nazbuhn@gmail.com (Corresponding Author)
†sadunur@calpoly.edu, jhamilt3@uccs.edu, slevis@uccs.edu, ghagen@uccs.edu, jventu09@calpoly.edu

Abstract

Background: Live-cell fluorescence microscopy enables the study of dynamic cellular processes. However, fluorescence microscopy can damage cells and disrupt these dynamic processes through photobleaching and phototoxicity. Reducing a sample's light exposure mitigates the effects of photobleaching and phototoxicity but results in low signal-to-noise ratio (SNR) images. Deep learning provides a solution for restoring these low-SNR images. However, these deep learning methods require large, representative datasets for training, testing, and benchmarking, as well as substantial GPU memory, particularly for denoising large images. **Results:** We present a new fluorescence microscopy dataset designed to expand the range of imaging conditions and specimens currently available for evaluating denoising methods. The dataset contains 17,568 paired high/low-SNR images across 15 sub-datasets that vary in specimen, imaging modality, objective, staining type, excitation wavelength, and exposure time. We evaluated five state-of-the-art deep learning denoising models on the dataset, including supervised, unsupervised, and zero-shot techniques. We also developed an image stitching method that enables large images to be processed in smaller crops and reconstructed. **Conclusions:** Our dataset provides a diverse benchmark for evaluating deep learning denoising methods, and our stitching method provides a solution to GPU memory constraints encountered when processing large images. Among the evaluated deep learning models, the supervised Transformer-based model had the best denoising performance but required the longest training time.

Key words: fluorescence microscopy; image restoration; deep learning; image stitching; phototoxicity; denoising

Background

The imaging of live cells and tissues is an essential process that enables scientists to observe dynamic cellular activity. Live imaging is commonly performed using fluorescence microscopy, which allows for the detection and tracking of biological molecules with high sensitivity and specificity [1]. These biological molecules are probed with fluorophores, which are excited by distinct wavelengths of light to produce emissions used to generate images. However, during the excitation of fluorophores, photobleaching and phototoxicity can occur, which introduce challenges to the consistency and reproducibility of imaging data [2].

The excitation of fluorophores can result in damage to their chemical structure, a process known as photobleaching. As fluorophores undergo photobleaching, they may interact with oxygen, generating reactive oxygen species (ROS) [2]. An unnatural increase in ROS can cause phototoxicity, leading to detrimental changes in a specimen, including damage to DNA, induced mutations, oxidized proteins, and potential disruption of the developmental processes within a cell [2].

Compiled on: June 5, 2026.
Draft manuscript prepared by the author.

To produce reliable data, phototoxicity must be minimized. Accordingly, numerous approaches have been developed to mitigate its effects. Many strategies focus on reducing a sample's light exposure by modifying microscope hardware, sample environment, and imaging conditions. One approach has been limiting light exposure to areas outside the focal plane. This approach serves as the basis for multiple fluorescence microscopy modalities, including total internal reflection fluorescence, lateral sheet fluorescence microscopy, and two-photon microscopy [3]. Despite these methods, phototoxicity remains a challenge, since high local light intensities can still damage cells, especially during prolonged live imaging [2].

Reducing excitation light intensity and/or exposure times can minimize photobleaching and phototoxicity but leads to low signal-to-noise ratio (SNR) images. To circumvent this, computational image restoration techniques have been applied to restore low SNR images. However, traditional restoration algorithms, such as BM3D, which rely on predefined mathematical models and heuristics, struggle to address complex noise patterns [4]. In contrast, deep learning models can learn the structure of complex noise types, including cases with unknown noise levels [5]. Recent work by Hagen et al. demonstrates that these deep learning models outperform traditional models in terms of quantitative metrics and visual quality [6].

Deep learning is a subset of machine learning that uses artificial neural networks, computational networks inspired by the brain, to learn patterns from large datasets. These networks rely on layers of interconnected nodes to transform input data into outputs using learned weights. Among deep learning methods, convolutional neural networks (CNNs) [7] have been proven effective for image-related tasks. CNNs use filters to extract image features like edges, textures, and shapes, allowing the network to learn visual patterns. More recently, Transformer-based models, initially developed for natural language processing [8], have been adapted for vision tasks [9], offering advantages in long-range dependencies and global contexts compared to CNNs.

Deep learning approaches for image restoration can be classified into three major categories: supervised, self-supervised, and zero-shot [10]. Supervised methods rely on large datasets of paired low and high SNR images to learn the relationship between noisy and clean images. In contrast, self-supervised methods do not rely on ground truth clean images, and instead learn denoising from noisy data alone. Zero-shot methods denoise a single noisy image without training on a representative dataset. Extensive evaluation datasets are required to benchmark the performance of supervised, self-supervised, and zero-shot methods.

To meet the data requirements necessary for denoising models, datasets should span fluorescence microscopy imaging modalities and biological specimens [11, 12, 6, 13, 14]. Such datasets should capture complex noise patterns arising from varying imaging conditions and specimens, enabling supervised models to learn robust denoising mappings. This range of conditions allows for the assessment of model generalizability and helps prevent overfitting to specific training data. These datasets serve as valuable benchmarks for comparing deep learning methods for fluorescence microscopy image denoising.

While dataset diversity is important for training and evaluating fluorescence microscopy denoising methods, few datasets are currently available. One such dataset, introduced by Zhang et al. [11], consists of 12,000 images spanning fluorescence microscopy modalities, including confocal, two-photon, and widefield. This dataset includes samples from cells, zebrafish, and mouse brain tissue, but is limited in both sample diversity and image quality. The W2S dataset introduced by Zhou et al. [12], provides 360 image sets with varying noise levels but is constrained to solely widefield imaging. Weigert et al. [15] provided a collection of paired image patches for two specimens to evaluate denoising methods. More recently, Hagen et al. [6] introduced a dataset consisting of 567 paired images ranging from 0.26 to 4.19 megapixels, covering both widefield and confocal imaging modalities. This dataset includes images of actin, mitochondria, nucleus, and membrane samples. However, it remains limited in terms of noise level variation, image sizes, and sample types. A well-suited dataset for training deep learning models should include a diverse range of fluorescence microscopy modalities, objective lenses, noise levels, exposure times, and biological samples.

We introduce a novel collection of 324 full-size, paired images, separated into 17,568 paired image crops, containing a greater diversity of specimens and more extreme noise levels than existing fluorescence microscopy denoising datasets. Previously unrepresented specimens include breast cancer tissue array spots, earthworm, freshwater fish gills, rat testis, rabbit testis, human ovary, and tubulin. Imaging modalities include widefield and spinning disk confocal microscopy. We include more extreme noise levels than in previous datasets by widening the gap between high and low exposure times. This increased diversity of specimens and noise conditions makes our dataset a more comprehensive and well-suited resource for evaluating the performance of fluorescence microscopy denoising models. More extreme noise poses a challenge for denoising methods, since the larger variance in the data makes it more difficult for such methods to determine the underlying clean signal from limited samples. Different specimen types pose a challenge due to the diversity of visual patterns and varying level of self-similarity in the samples.

In addition to the necessity of large, diverse datasets, deep learning approaches require substantial memory for computation. As a result, denoising of fluorescence microscopy images is limited by GPU memory constraints. To address this challenge, we introduce an image stitching approach that enables large images to be denoised in smaller crops and reassembled [16, 17, 18, 19]. Inspired by panorama stitching techniques [20, 21] we optimize per-tile brightness and contrast terms to compensate for the output variation introduced by learning-based denoising methods, and apply linear blending in the overlap region.

We evaluated the performance of five state-of-the-art deep learning denoising models using our dataset. Our selection included supervised [15, 22], unsupervised [23, 24], and zero-shot [25] methods. BM3D [4] was excluded from our analysis due to its inferior performance compared to CARE [15] in a previous study [6]. Self-Supervised Poisson Gaussian Denoising (SSPG) [23] is an extension of blindspot denoising techniques [26, 27] to support the Poisson-Gaussian noise model which is commonly applied in fluorescence microscopy. Self-Inspired Noise2Noise (SN2N) [24] employs an interpolation technique to generate data for Noise2Noise [28] training. SSPG and SN2N are self-supervised techniques to learn a denoiser from noisy data alone. Noise2Fast, proposed by Lequyer et al. [25], is a zero-shot unsupervised denoising method, meaning that it does not require any training on a separate dataset, and instead learns to denoise directly from a single input image. Noise2Fast relies on a checkerboard-down sampling technique to generate a set of four images from the input noisy image, which are used to train a lightweight feed-forward neural network using Noise2Noise-style self-supervision [28]. Content Aware Image Restoration (CARE), developed by Weigert et al. [15], is a supervised model that utilizes a U-Net architecture [29, 30] to learn mappings from degraded images to denoised versions. Restormer, introduced by Zamir et al. [22], is a supervised denoising model that employs an encoder-decoder Transformer-based architecture. The model's core components include a Multi-Dconv Head Transposed Attention (MDTA) block and a Gated Dconv Feed-Forward Network (GDFN). MDTA aids the model in learning both fine details and broader patterns by combining attention mechanisms and depth-wise convolutions. GDFN improves feature quality by using gates and convolutional layers to refine and enhance each layer's image content. Transformer-based models have outperformed the more commonly-used U-Net in previous studies [31].

Table 1. Overview of the samples and preparations in our dataset.

Dataset(s)	Sample Type	Stain	Sample Source	Catalog Number
01	Actin	Alexa488-pahlloidin	Invitrogen	Fluoroslides #1
02	Breast Cancer Array	H&E Stain	Tissuearray.com	BR249
03	Earthworm	H&E Stain	Eisco	BS18225
04 – 06	Fish Gill	H&E Stain	Eisco	BS18101
07 – 08	Rat Testis	H&E Stain	Carolina	316464
09 – 10	Human Ovary	H&E Stain	Carolina	616024
11	Immature Ovary	H&E Stain	Eisco	BS18222
12	Mitochondria	MitoTracker Red	Invitrogen	Fluoroslides #1
13	Mouse Brain	GFP	Sunjin Lab	-
14	Rabbit Testis	H&E Stain	Amscope	PS50
15	Tubulin	BODIPY FL-GAM	Invitrogen	Fluoroslides #2

Table 2. Overview of dataset imaging conditions. WF indicates widefield and CF indicates spinning disk confocal.

Dataset	Type	No. Images	Sizes [MP]	No. Crops	Exp. Time (Low / High) [ms]	Obj. Mag./NA	Ex. [nm]	Em. [nm]	a	b
01	WF	23	5 - 54.86	1,552	1 / 100	60×/1.42	470	525	0.0092	0.0003
02	WF	1	282	1,024	1 / 100	60×/1.42	530	575	0.0091	-0.0007
03	WF	1	134	400	1 / 600	20×/0.45	530	575	0.0321	-0.0116
04	WF	1	256	576	1 / 100	20×/0.45	530	575	0.0116	-0.0009
05	WF	12	19 - 68	1,328	1 / 300	10×/0.40	530	575	0.0419	-0.0152
06	WF	11	28 -55	1,344	1 / 600	10×/0.40	530	575	0.0385	-0.0148
07	CF	105	4	1,680	20 / 200	60×/1.35	532	575	0.0023	-0.0001
08	CF	100	4	1,600	20 / 500	60 ×/1.35	450	575	0.0146	-0.0032
09	WF	13	28 - 72	1,424	1 / 100	10×/0.4	530	575	0.0171	-0.0028
10	WF	11	28 - 55	1,344	1 / 100	4×/0.16	530	575	0.0177	-0.0025
11	WF	1	90	256	1 / 600	20×/0.45	530	575	0.0356	-0.0123
12	WF	14	5 - 55	1,568	1 / 100	60×/1.42	530	575	0.0197	-0.0048
13	WF	8	36-126	1,216	1 / 100	10×/0.4	470	515	0.0327	-0.0095
14	WF	9	5 - 81	928	1 / 100	10×/0.40	530	575	0.0137	-0.0015
15	WF	14	5 - 55	1,328	1 / 100	60×/1.42	470	515	0.0215	-0.0045

Data Description

We generated fifteen distinct datasets of paired low and high-SNR images composed of specimens from actin, breast cancer array, earthworm, fish gill, rat testes, immature ovaries, human ovaries in active phase, mitochondria, mouse brain, rabbit testes, and tubulin as detailed Table 1. There are 324 images in total, ranging in size from 4.19 to 282.22 megapixels (MP). Imaging conditions varied in light intensity, exposure times, objectives, excitation wavelengths, and stains as detailed in Table 2. We extracted non-overlapping crops of size 512 × 512 pixels from the images, resulting in a total of 17,568 crops, which we then divided into a 90%/10% train/test split for each dataset.

We estimated the noise level of the images using the single-image Poisson-Gaussian noise estimation method of Foi et al. [32]. This approach models the variance of the noisy pixel value z in terms of the clean value x as $\mathrm{Var}[z] = ax + b$, where a and b are the parameters of the model. We estimated the noise parameters for each image crop and report the average values for each dataset in Table 2. Compared to the Fluorescence Microscopy Dataset (FMD) [11], our dataset has similar levels of Poisson noise (a) but generally an order of magnitude higher Gaussian noise (b).

We also estimated the noise level of the images by calculating the PSNR of the raw, low-SNR (noisy) image compared to the high-SNR (clean) image (see Table 3). The average raw PSNR of our datasets ranges from 14.12 to 27.66 dB, with an average of 19.92 dB. For comparison, the raw PSNR in the dataset of Hagen et al. [6] ranged from 18.34 to 29.4 dB, with an average of 24.34 dB, and the FMD dataset [11] had an average raw PSNR of 27.22 dB. Thus our dataset consists of more extreme low-SNR data and tests a previously unexplored boundary of what current denoising methods can handle.

Analyses

Denoising performance. The denoising performance of each method is summarized in Table 3 and visualized in Figure 3. Figures 1 and 2 shows representative low-SNR input images, denoised outputs from each model, and the corresponding high-SNR ground truth images from each dataset, with the "fire" colormap applied to enhance contrast. Restormer consistently achieved the highest average PSNR and SSIM. CARE and Noise2Fast's comparative performance varied depending on the dataset. The performance of the unsupervised techniques SSPG and SN2N was typically behind the other methods.

Speed. Restormer's training times ranged from 16-18 hours to several days, while the CARE network trained in approximately 3.5 hours per dataset, SSPG about 1 hour per dataset, and SN2N about 8.5 hours per dataset. Noise2Fast does not require training on a separate training set. Inference times also varied between models: Restormer required about 0.45 seconds per image, CARE about 1 second per image, SSPG about 0.20 seconds per image, SN2N about 0.08 seconds per image, and Noise2Fast, due to its iterative self-supervised denoising method, between 9 and 40 seconds per image.

Adaptive stitching. The deep learning models introduced variations in light intensity between neighboring denoised crops, as shown in Figure 4. Our adaptive image stitching method reduces these intensity differences across stitched crops compared to naïve stitching. Figure 5 shows an example adaptively stitched Restormer result compared to the corresponding low and high SNR images.

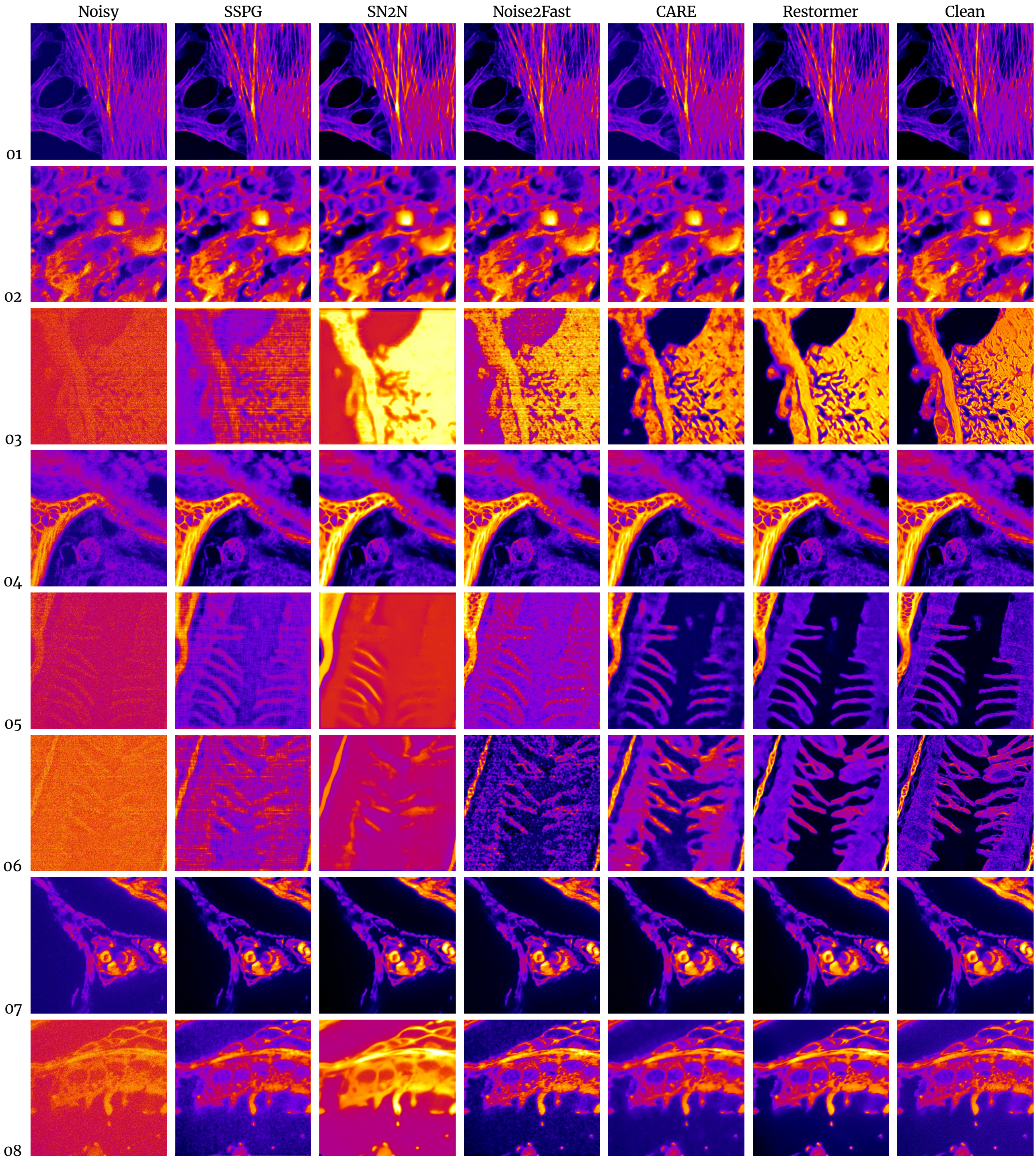

Figure 1. Example noisy input, denoised, and clean images.

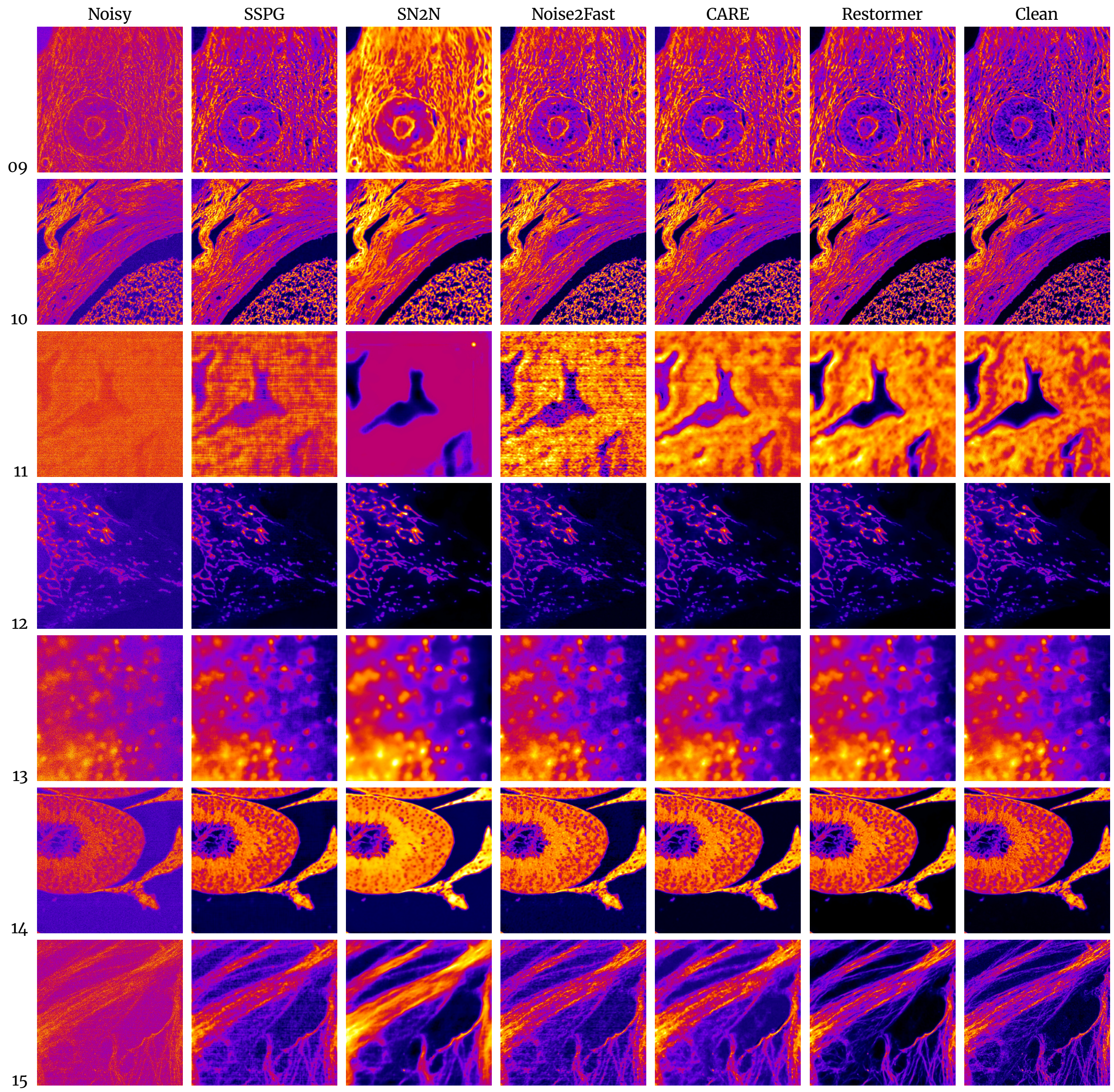

Figure 2. Example noisy input, denoised, and clean images.

Table 3. Average test set results. The highest values per dataset are in bold.

	PSNR						SSIM					
Dataset	Noisy	SSPG	SN2N	Noise2Fast	CARE	Restormer	Noisy	SSPG	SN2N	Noise2Fast	CARE	Restormer
01	23.22	29.05	28.59	29.47	29.50	**31.01**	0.37	0.63	0.63	0.65	0.65	**0.68**
02	23.31	29.83	29.64	30.15	28.07	**34.26**	0.40	0.77	0.77	0.78	0.74	**0.82**
03	17.15	17.68	18.59	18.62	18.54	**19.89**	0.08	0.10	0.13	0.15	0.12	**0.18**
04	22.32	32.17	30.55	32.38	32.04	**33.41**	0.39	0.84	0.80	0.85	0.85	**0.87**
05	15.70	18.51	20.02	20.02	20.83	**23.90**	0.05	0.13	0.29	0.23	0.28	**0.43**
06	15.40	17.80	19.42	19.14	20.33	**23.16**	0.05	0.12	0.27	0.20	0.28	**0.42**
07	27.66	29.51	29.61	29.37	30.28	**33.73**	0.75	0.88	0.88	0.88	0.89	**0.93**
08	18.09	24.52	26.05	26.60	26.60	**27.64**	0.14	0.43	0.47	0.49	0.49	**0.53**
09	19.78	24.80	23.63	25.94	25.88	**26.80**	0.34	0.61	0.49	0.65	0.64	**0.68**
10	21.14	26.25	24.33	26.83	27.03	**28.12**	0.43	0.64	0.55	0.66	0.68	**0.72**
11	14.12	16.21	17.33	17.41	18.06	**21.43**	0.03	0.08	0.22	0.16	0.22	**0.39**
12	22.86	29.42	29.20	30.44	30.38	**32.16**	0.30	0.63	0.63	0.69	0.70	**0.75**
13	18.35	24.01	26.08	25.71	24.43	**28.24**	0.11	0.35	0.46	0.42	0.37	**0.50**
14	21.21	29.07	27.82	29.24	29.35	**30.60**	0.39	0.75	0.71	0.76	0.77	**0.79**
15	18.56	25.99	26.12	27.07	27.15	**28.73**	0.16	0.51	0.53	0.57	0.60	**0.66**

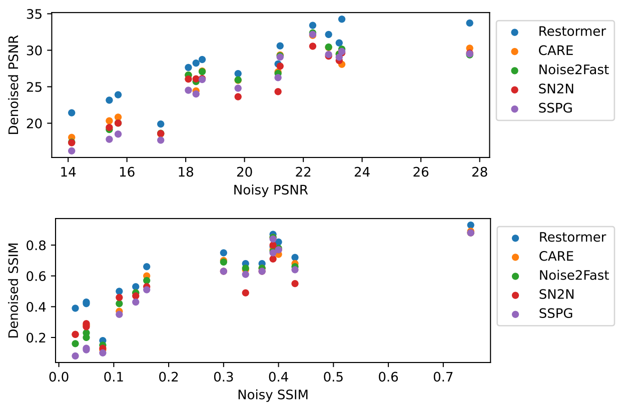

Figure 3. Average noisy vs. denoised PSNR and SSIM for each model and dataset.

Discussion

Across the specimens, imaging modalities, and noise conditions present in our dataset, Restormer achieved the highest performance in terms of PSNR and SSIM. However, due to its supervised training and Transformer-based encoder-decoder architecture, Restormer required substantially longer training times than other supervised models.

We found the denoising performance of SSPG and SN2N to be behind the other methods, which is reasonable considering that the supervised methods (CARE and Restormer) have access to clean data during training, and the zero-shot method (Noise2Fast) trains directly on the test image. Additionally, SSPG, SN2N, and Noise2Fast all make the assumption of independent and identically distributed (i.i.d.) noise, whereas our dataset contains structured noise. Thus the unsupervised and zero-shot methods we tested are likely hindered by the i.i.d. noise assumption.

While its computational costs were considerably higher, Restormer was the most effective model for removing noise while preserving the structural integrity of the original images. Preserving the fine structural detail of fluorescence microscopy images is particularly important because the introduction of artifacts compromises the reliability of downstream biological analysis. This highlights the critical trade-off between computational cost and model accuracy in fluorescence microscopy denoising.

In addition to computational requirements, supervised deep learning models rely on representative, paired image datasets to learn robust mappings from low-SNR to high-SNR images. Denoising performance depends on a model's ability to learn these mappings, but with limited or unrepresentative training datasets, models risk overfitting to specific imaging conditions and fail to handle the inherent variability of live fluorescence microscopy imaging. In contrast, unsupervised approaches do not require paired training data, but they demonstrated inferior denoising performance, as they struggled to remove noise and maintain the structural integrity of the images in our dataset.

The deep learning models introduced variations in light intensity between the individually denoised neighboring crops, resulting in visible seams and uneven lighting in the reconstructed images. To address the issue of uneven lighting in denoised crops, we implemented an adaptive stitching approach that adjusts each image segment's light intensity based on its neighbors. This method was able to effectively decrease intensity variation across images. Our adaptive stitching process enabled the denoising of large images using deep learning models without exceeding our memory constraints.

Potential implications

Our results highlight the relationship between computational costs and denoising accuracy, underscoring the need for deep learning denoising models that are both computationally efficient and able to remove noise without compromising image integrity. Additionally, given the diversity of biological structures included in our dataset, it would be valuable to explore whether certain deep learning techniques are more effective for denoising specific biological structures. Lastly, since tiny structural distortions can have a significant impact on biological interpretations but may not strongly affect PSNR and SSIM, there is a need for image quality metrics specifically tailored to microscopy images [33].

Methods

Microscopy. We acquired the fluorescence images using an Olympus BX53 microscope equipped with a motorized XY stage (Applied Scientific Imaging, Eugene, OR), Aura III light source (Lumencor, Beaverton, OR), Fluorescence filters (Chroma, Bellows Falls, VT), and Fusion BT camera (Hamamatsu Photonics, Hamamatsu, Japan). The spinning disk confocal setup is described in our previous work [34].

Deep learning methods. Each model was trained and tested using an Nvidia V100 32GB GPU. For each method we used the authors' provided implementations and default settings for training parameters such as loss function, batch size, and learning rate schedule.

For fair comparison, we used consistent data pre-processing and data augmentation procedures across all methods to the extent possible. For pre-processing during training, the images were normalized using percentile normalization. For data augmentation, we horizontally and vertically flipped the images and rotated by 90, 180, and 270 degrees. SSPG, SN2N, CARE and Restormer were trained on patches randomly sampled from the crops. For SSPG, SN2N, and CARE we used 128×128 pixel patches. The recommended approach for Restormer is to increase patch size during training; we began with 128×128 pixel patches and increased to 384×384 pixels by the end of training. Noise2Fast has its own training procedure specific to its efficient and zero-shot design, and so does not use patching or data augmentation.

Image quality metrics. We analyzed the performance of the models on a holdout test set from each dataset not used during training. Performance was quantified using peak signal-to-noise ratio (PSNR) and structural similarity index measure (SSIM) [35] by comparing each denoised result to its corresponding ground truth image. PSNR measures the ratio of the maximum signal power in an image to the power of the noise present in the image. SSIM is based on human visual perception of an image and evaluates image similarity using contrast, luminance, and structure. SSIM values range from zero to one. Higher PSNR and SSIM values indicate increased image similarity and, thus, greater denoising success.

Prior to calculating PSNR and SSIM, images were normalized using the "minimum MSE" normalization method ([15], Supplementary Notes, Section 2.2).

Adaptive image stitching. We prepared the full-size test images for stitching by adding 64-pixel reflective padding around the borders and then dividing the images into 640×640 pixel overlapping crops, so that each crop overlapped with its neighbors by 64 pixels, as illustrated in Figure 6. The central region of each crop, excluding the overlap, represents the valid region of 512×512 pixels.

Crops were denoised using the trained Restormer model, then processed in our adaptive stitching algorithm to re-assemble the crops back into a full-size composite denoised image. We optimized the agreement between overlapping regions by adjusting

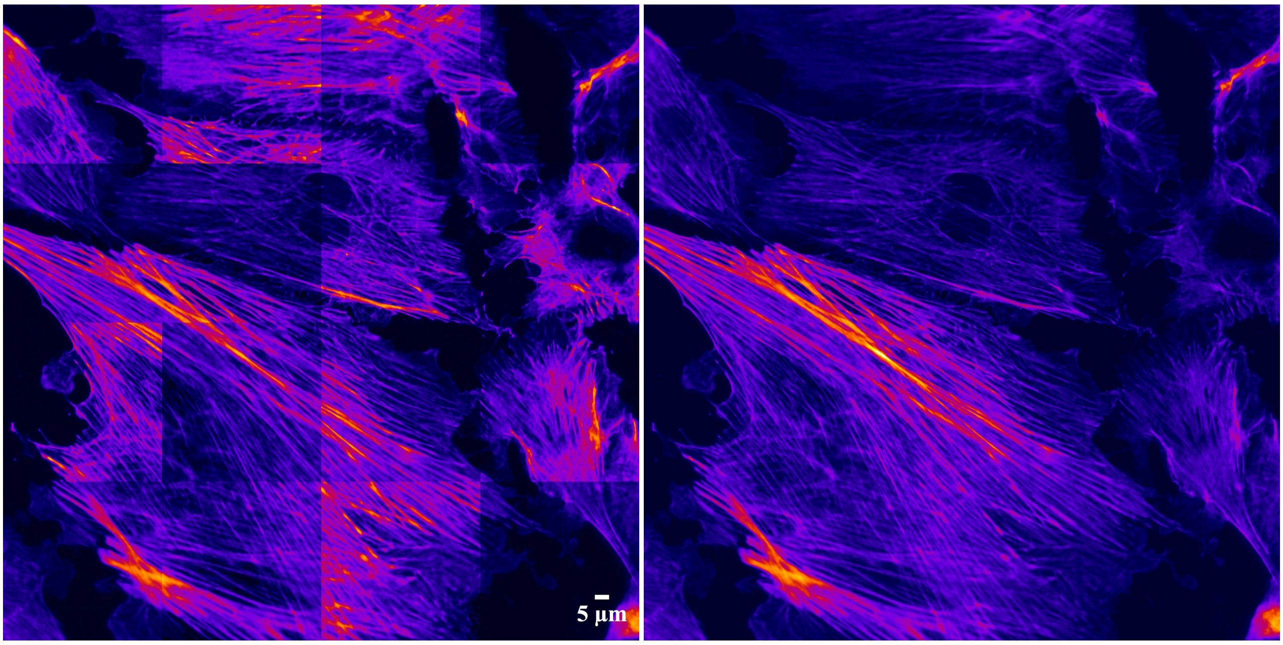

(a) Naïve Stitching **(b)** Adaptive Stitching

Figure 4. Effect of stitching logic on Restormer-denoised images from Dataset 1. (a) Naïve stitching of non-overlapping crops. (b) Adaptive stitching of overlapping crops. Final images are 2048×2048 pixels.

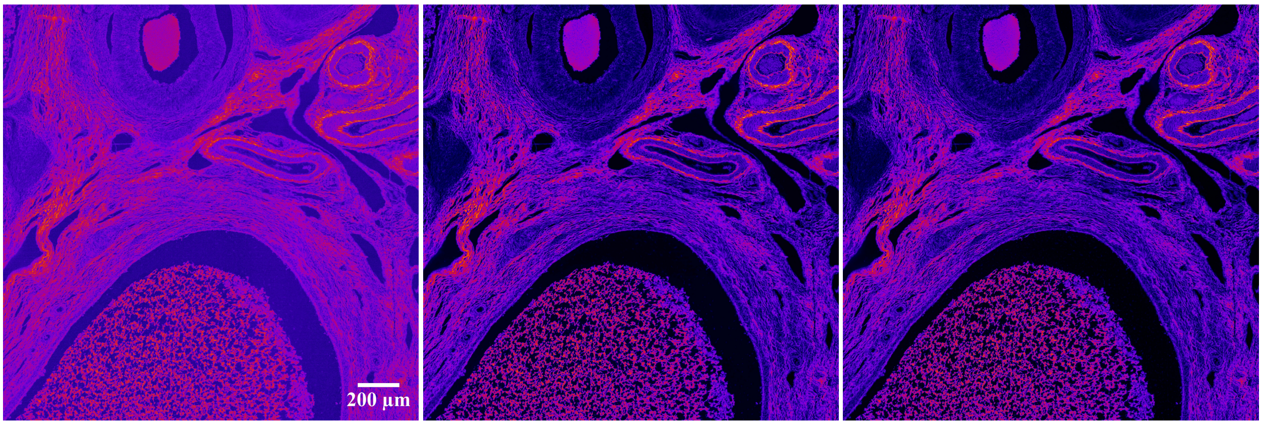

(a) Noisy **(b)** Denoised **(c)** Clean

Figure 5. Example adaptively stitched image from Dataset 9. (a) Noisy image. (b) Adaptive stitching of Restormer denoising results. (c) Clean image. All images are 3072×3072 pixels.

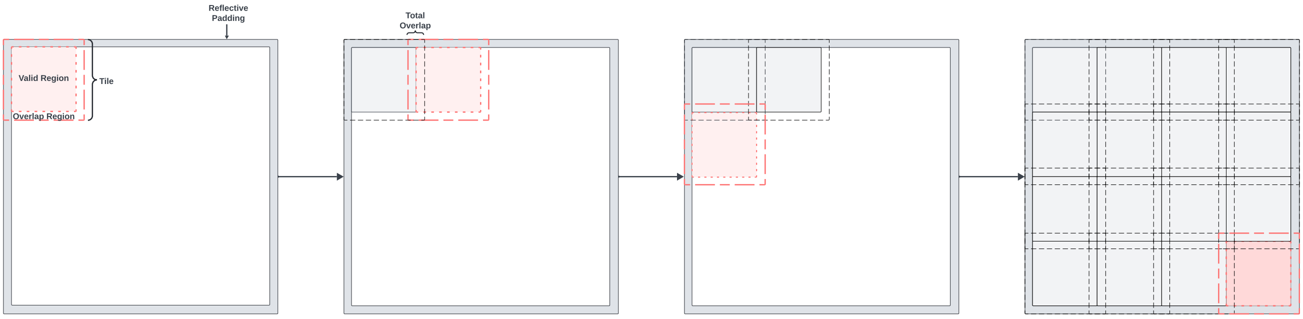

Figure 6. Image cropping logic. The red tile shows the progression of the cropping logic. The shaded area marks the valid region of each tile, while the overlapping region is the space between the valid region and the outer dashed line. Cropping logic proceeds until the entire image is processed.

each crop's scale and shift using the trust region reflective algorithm over a least squares objective. To anchor the adaptive stitching algorithm, we identified the noisy crop with the greatest intensity and assigned its corresponding crop in the denoised image a fixed scale and shift. Following intensity adjustment, a feathered weight mask was applied to each crop, with the weight linearly decreasing from the center of each crop to its edges. In overlapping regions, pixels from all neighboring crops were summed and divided by the total weight at each position, producing smooth transitions between crops.

Availability of source code and requirements

- Project name: Comparison of Deep Learning Approaches for Extreme Low-SNR Image Restoration software and analytics code
- Project home page: https://github.com/nazbuhn/extreme-low-snr-restoration
- Operating systems(s): Platform independent
- Programming language(s): Python
- License: MIT License

Data availability

All raw and analyzed data [36] is available on GigaDB [37]. All files and data are distributed under the Creative Commons CC0 waiver, with a request for attribution. DOME-ML (Data, Optimisation, Model, and Evaluation in Machine Learning) annotation supporting the current study is available through DOME Registry [38].

Declarations

List of abbreviations

BM3D: block matching and 3D filtering; CARE: content-aware image restoration; CNN: convolutional neural network; GDFN: Gated-Dcov Feed-Forward Network; GPU: graphics processing unit; MDTA: multi-Dconv transposed attention; MP: megapixels; NA: numerical aperture; PSNR: peak signal-to-noise ratio; ROS: reactive oxygen species; SN2N: Self-Inspired Noise2Noise; SNR: signal to noise ratio; SSIM: structural similarity index measure; SSPG: Self-Supervised Poisson-Gaussian Denoising

Ethical Approval

Not applicable

Consent for publication

Not applicable

Competing Interests

The authors declare that they have no competing interests.

Funding

This work was supported by the National Institute of General Medical Sciences of the National Institutes of Health under award number 2R15GM128166-02. This work was also supported by the UCCS BioFrontiers Center. The funding sources had no involvement in study design; in the collection, analysis and interpretation of data; in the writing of the report; or in the decision to submit the article for publication.

Author's Contributions

N.E.B: investigation, methodology, software, validation, visualization, writing – original draft, writing – review & editing; S.R.A.: investigation, methodology, software, validation, visualization, writing – original draft; J.H.: data curation; SL: data curation; G.M.H.: conceptualization, data curation, funding acquisition, project administration, resources, supervision, validation, writing – review & editing; J.D.V.: conceptualization, funding acquisition, project administration, software, supervision, validation, writing - review & editing

Acknowledgements

Not applicable

References

1. Pylvänäinen JW, Gómez-de Mariscal E, Henriques R, Jacquemet G. Live-cell imaging in the deep learning era. Current Opinion in Cell Biology 2023;85:102271. 10.1016/j.ceb.2023.102271.
2. Icha J, Weber M, Waters JC, Norden C. Phototoxicity in live fluorescence microscopy, and how to avoid it. BioEssays 2017;39(8):1700003. 10.1002/bies.201700003.
3. Fritzky L, Lagunoff D. Advanced methods in fluorescence microscopy. Analytical Cellular Pathology 2013;36(1-2):5–17. 10.3233/ACP-120071.
4. Dabov K, Foi A, Katkovnik V, Egiazarian K. Image denoising by sparse 3-D transform-domain collaborative filtering. IEEE Transactions on Image Processing 2007;16(8):2080–2095. 10.1109/TIP.2007.901238.
5. Zhang K, Zuo W, Chen Y, Meng D, Zhang L. Beyond a Gaussian denoiser: Residual learning of deep CNN for image denoising. IEEE Transactions on Image Processing 2017;26(7):3142–3155. 10.1109/TIP.2017.2662206.
6. Hagen GM, Bendesky J, Machado R, Nguyen TA, Kumar T, Ventura J. Fluorescence microscopy datasets for training deep neural networks. GigaScience 2021;10(5):giab032. 10.1093/gigascience/giab032.
7. LeCun Y, Bottou L, Bengio Y, Haffner P. Gradient-based learning applied to document recognition. Proceedings of the IEEE 2002;86(11):2278–2324. 10.1109/5.726791.
8. Vaswani A, Shazeer N, Parmar N, Uszkoreit J, Jones L, Gomez AN, et al. Attention is all you need. Advances in Neural Information Processing Systems 2017;30.
9. Dosovitskiy A, Beyer L, Kolesnikov A, Weissenborn D, Zhai X, Unterthiner T, et al. An image is worth 16x16 words: Transformers for image recognition at scale. In: International Conference on Learning Representations; 2021. .
10. Elad M, Kawar B, Vaksman G. Image denoising: The deep learning revolution and beyond—a survey paper. SIAM Journal on Imaging Sciences 2023;16(3):1594–1654. 10.1137/23M1545859.
11. Zhang Y, Zhu Y, Nichols E, Wang Q, Zhang S, Smith C, et al. A Poisson-Gaussian denoising dataset with real fluorescence microscopy images. In: Proceedings of the IEEE/CVF Conference on Computer Vision and Pattern Recognition; 2019. p. 11710–11718. 10.1109/CVPR.2019.01198.
12. Zhou R, El Helou M, Sage D, Laroche T, Seitz A, Süsstrunk S. W2S: Microscopy data with joint denoising and super-resolution for widefield to SIM mapping. In: European Conference on Computer Vision Springer; 2020. p. 474–491. 10.1007/978-3-030-66415-2_31.
13. Qiao C, Li D, Guo Y, Liu C, Jiang T, Dai Q, et al. Evaluation and de-

velopment of deep neural networks for image super-resolution in optical microscopy. Nature Methods 2021;18(2):194–202. 10.1038/s41592-020-01048-5.
14. Venkataramanan A, Kloster M, Burfeid-Castellanos A, Dani M, Mayombo NA, Vidakovic D, et al. "UDE DIATOMS in the Wild 2024"': a new image dataset of freshwater diatoms for training deep learning models. GigaScience 2024;13:giae087. 10.1093/gigascience/giae087.
15. Weigert M, Schmidt U, Boothe T, Müller A, Dibrov A, Jain A, et al. Content-aware image restoration: Pushing the limits of fluorescence microscopy. Nature Methods 2018;15(12):1090–1097. 10.1038/s41592-018-0216-7.
16. Rankov V, Locke RJ, Edens RJ, Barber PR, Vojnovic B. An algorithm for image stitching and blending. In: Three-dimensional and Multidimensional Microscopy: Image Acquisition and Processing XII, vol. 5701 SPIE; 2005. p. 190–199. 10.1117/12.590536.
17. Preibisch S, Saalfeld S, Tomancak P. Globally optimal stitching of tiled 3D microscopic image acquisitions. Bioinformatics 2009;25(11):1463–1465. 10.1093/bioinformatics/btp184.
18. Legesse FB, Chernavskaia O, Heuke S, Bocklitz T, Meyer T, Popp J, et al. Seamless stitching of tile scan microscope images. Journal of Microscopy 2015;258(3):223–232. 10.1111/jmi.12236.
19. He B, Zhang Y, Zhang Z, Cheng Y, Zhang F, Sun F, et al. vEMstitch: an algorithm for fully automatic image stitching of volume electron microscopy. GigaScience 2024;13:giae076. 10.1093/gigascience/giae076.
20. Brown M, Lowe DG. Automatic panoramic image stitching using invariant features. International Journal of Computer Vision 2007;74(1):59–73. 10.1007/s11263-006-0002-3.
21. Ma B, Zimmermann T, Rohde M, Winkelbach S, HeFeng FQ, Lindenmaier W, et al. Use of Autostitch for automatic stitching of microscope images. Micron 2007;38(5):492–499. 10.1016/j.micron.2006.07.027.
22. Zamir SW, Arora A, Khan S, Hayat M, Khan FS, Yang MH. Restormer: Efficient Transformer for high-resolution image restoration. In: Proceedings of the IEEE/CVF Conference on Computer Vision and Pattern Recognition; 2022. p. 5728–5739. 10.1109/CVPR52688.2022.00564.
23. Khademi W, Rao S, Minnerath C, Hagen G, Ventura J. Self-supervised Poisson-Gaussian denoising. In: Proceedings of the IEEE/CVF Winter Conference on Applications of Computer Vision; 2021. p. 2131–2139. 10.1109/WACV48630.2021.00218.
24. Qu L, Zhao S, Huang Y, Ye X, Wang K, Liu Y, et al. Self-inspired learning for denoising live-cell super-resolution microscopy. Nature Methods 2024;21(10):1895–1908. 10.1038/s41592-024-02400-9.
25. Lequyer J, Philip R, Sharma A, Hsu WH, Pelletier L. A fast blind zero-shot denoiser. Nature Machine Intelligence 2022;4(11):953–963. 10.1038/s42256-022-00547-8.
26. Krull A, Buchholz TO, Jug F. Noise2Void - Learning denoising from single noisy images. In: Proceedings of the IEEE/CVF Conference on Computer Vision and Pattern Recognition; 2019. p. 2129–2137. 10.1109/CVPR.2019.00223.
27. Laine S, Karras T, Lehtinen J, Aila T. High-quality self-supervised deep image denoising. Advances in Neural Information Processing Systems 2019;32.
28. Lehtinen J, Munkberg J, Hasselgren J, Laine S, Karras T, Aittala M, et al. Noise2Noise: Learning Image Restoration without Clean Data. In: International Conference on Machine Learning PMLR; 2018. p. 2965–2974.
29. Ronneberger O, Fischer P, Brox T. U-Net: Convolutional networks for biomedical image segmentation. In: International Conference on Medical Image Computing and Computer-Assisted Intervention Springer; 2015. p. 234–241. 10.1007/978-3-319-24574-4_28.
30. Falk T, Mai D, Bensch R, Çiçek Ö, Abdulkadir A, Marrakchi Y, et al. U-Net: deep learning for cell counting, detection, and morphometry. Nature Methods 2019;16(1):67–70. 10.1038/s41592-018-0261-2.
31. Shah ZH, Müller M, Hübner W, Wang TC, Telman D, Huser T, et al. Evaluation of Swin Transformer and knowledge transfer for denoising of super-resolution structured illumination microscopy data. GigaScience 2024;13:giad109. 10.1093/gigascience/giad109.
32. Foi A, Trimeche M, Katkovnik V, Egiazarian K. Practical Poissonian-Gaussian noise modeling and fitting for single-image raw-data. IEEE Transactions on Image Processing 2008;17(10):1737–1754. 10.1109/TIP.2008.2001399.
33. Gunawan I, Marsh RJ, Aggarwal N, Meijering E, Cox S, Lock JG, et al. Image quality metrics fail to accurately represent biological information in fluorescence microscopy. bioRxiv 2025;p. 2025–08. 10.1101/2025.08.05.668508.
34. Hagen GM, Lewis B, Levis S, Hamilton JR, Paul TC. Low-Cost Spinning Disk Confocal Microscopy with a 25-Megapixel Camera. Sensors 2025;25(23):7183. 10.3390/s25237183.
35. Wang Z, Bovik AC, Sheikh HR, Simoncelli EP. Image quality assessment: from error visibility to structural similarity. IEEE Transactions on Image Processing 2004;13(4):600–612. 10.1109/TIP.2003.819861.
36. Buhn NE, Adunur SR, Hamilton J, Levis S, Hagen GM, Ventura JD, Supporting Data for "Comparison of Deep Learning Approaches for Extreme Low-SNR Image Restoration"; 2026. GigaScience Database. https://doi.org/10.5524/102787.
37. Li X, Hua C, Yue Q, Li Z, Tong J, Luo Z, et al. GigaDB: A redesigned repository for data publishing and management. GigaScience 2026;p. giag047. 10.1093/gigascience/giag047.
38. Buhn NE, Adunur SR, Hamilton J, Levis S, Hagen GM, Ventura JD, Comparison of Deep Learning Approaches for Extreme Low-SNR Image Restoration [DOME-ML-Annotations]; 2026. DOME-ML Registry, https://registry.dome-ml.org/review/nry3lgt3c9.

Manuscript
Click here to access/download;Manuscript;Gigascience_2026_with_highlight

Placeholder for OUP logo oup.pdf

GigaScience, 2023, 1–9

doi: xx.xxxx/xxxx
Manuscript in Preparation
Research

RESEARCH

Comparison of Deep Learning Approaches for Extreme Low-SNR Image Restoration

Nasreen Elizabeth Buhn[1,*], Sriya Reddy Adunur[2,†], Joseph Hamilton[3,†], Summer Levis[3,†], Guy M. Hagen[3,†] and Jonathan D. Ventura[2,†]

[1]Biological Sciences Department, California Polytechnic State University, San Luis Obispo, California, 93407 and [2]Department of Computer Science and Software Engineering, California Polytechnic State University, San Luis Obispo, California, 93407 and [3]UCCS BioFrontiers Center, University of Colorado at Colorado Springs, 1420 Austin Bluffs Parkway, Colorado Springs, Colorado, 80918

[*]nazbuhn@gmail.com (Corresponding Author)
[†]sadunur@calpoly.edu, jhamilt3@uccs.edu, slevis@uccs.edu, ghagen@uccs.edu, jventu09@calpoly.edu

Abstract

Background: Live-cell fluorescence microscopy enables the study of dynamic cellular processes. However, fluorescence microscopy can damage cells and disrupt these dynamic processes through photobleaching and phototoxicity. Reducing a sample's light exposure mitigates the effects of photobleaching and phototoxicity but results in low signal-to-noise ratio (SNR) images. Deep learning provides a solution for restoring these low-SNR images. However, these deep learning methods require large, representative datasets for training, testing, and benchmarking, as well as substantial GPU memory, particularly for denoising large images. **Results:** We present a new fluorescence microscopy dataset designed to expand the range of imaging conditions and specimens currently available for evaluating denoising methods. The dataset contains 17,568 paired high/low-SNR images across 15 sub-datasets that vary in specimen, imaging modality, objective, staining type, excitation wavelength, and exposure time. We evaluated six state-of-the-art deep learning denoising models on the dataset, including supervised, unsupervised, and zero-shot techniques. We also developed an image stitching method that enables large images to be processed in smaller crops and reconstructed. **Conclusions:** Our dataset provides a diverse benchmark for evaluating deep learning denoising methods, and our stitching method provides a solution to GPU memory constraints encountered when processing large images. Among the evaluated deep learning models, the supervised Transformer-based model had the best denoising performance but required the longest training time.

Key words: fluorescence microscopy; image restoration; deep learning; image stitching; phototoxicity; denoising

Background

The imaging of live cells and tissues is an essential process that enables scientists to observe dynamic cellular activity. Live imaging is commonly performed using fluorescence microscopy, which allows for the detection and tracking of biological molecules with high sensitivity and specificity [1]. These biological molecules are probed with fluorophores, which are excited by distinct wavelengths of light to produce emissions used to generate images. However, during the excitation of fluorophores, photobleaching and phototoxicity can occur, which introduce challenges to the consistency and reproducibility of imaging data [2].

The excitation of fluorophores can result in damage to their chemical structure, a process known as photobleaching. As fluorophores undergo photobleaching, they may interact with oxygen, generating reactive oxygen species (ROS) [2]. An unnatural increase in ROS can cause phototoxicity, leading to detrimental changes in a specimen, including damage to DNA, induced mutations, oxidized proteins, and potential disruption of the developmental processes within a cell [2].

Compiled on: May 8, 2026.
Draft manuscript prepared by the author.

To produce reliable data, phototoxicity must be minimized. Accordingly, numerous approaches have been developed to mitigate its effects. Many strategies focus on reducing a sample's light exposure by modifying microscope hardware, sample environment, and imaging conditions. One approach has been limiting light exposure to areas outside the focal plane. This approach serves as the basis for multiple fluorescence microscopy modalities, including total internal reflection fluorescence, lateral sheet fluorescence microscopy, and two-photon microscopy [3]. Despite these methods, phototoxicity remains a challenge, since high local light intensities can still damage cells, especially during prolonged live imaging [2].

Reducing excitation light intensity and/or exposure times can minimize photobleaching and phototoxicity but leads to low signal-to-noise ratio (SNR) images. To circumvent this, computational image restoration techniques have been applied to restore low SNR images. However, traditional restoration algorithms, such as BM3D, which rely on predefined mathematical models and heuristics, struggle to address complex noise patterns [4]. In contrast, deep learning models can learn the structure of complex noise types, including cases with unknown noise levels [5]. Recent work by Hagen et al. demonstrates that these deep learning models outperform traditional models in terms of quantitative metrics and visual quality [6].

Deep learning is a subset of machine learning that uses artificial neural networks, computational networks inspired by the brain, to learn patterns from large datasets. These networks rely on layers of interconnected nodes to transform input data into outputs using learned weights. Among deep learning methods, convolutional neural networks (CNNs) [7] have been proven effective for image-related tasks. CNNs use filters to extract image features like edges, textures, and shapes, allowing the network to learn visual patterns. More recently, Transformer-based models, initially developed for natural language processing [8], have been adapted for vision tasks [9], offering advantages in long-range dependencies and global contexts compared to CNNs.

Deep learning approaches for image restoration can be classified into three major categories: supervised, self-supervised, and zero-shot [10]. Supervised methods rely on large datasets of paired low and high SNR images to learn the relationship between noisy and clean images. In contrast, self-supervised methods do not rely on ground truth clean images, and instead learn denoising from noisy data alone. Zero-shot methods denoise a single noisy image without training on a representative dataset. Extensive evaluation datasets are required to benchmark the performance of supervised, self-supervised, and zero-shot methods.

To meet the data requirements necessary for denoising models, datasets should span fluorescence microscopy imaging modalities and biological specimens [11, 12, 6, 13, 14]. Such datasets should capture complex noise patterns arising from varying imaging conditions and specimens, enabling supervised models to learn robust denoising mappings. This range of conditions allows for the assessment of model generalizability and helps prevent overfitting to specific training data. These datasets serve as valuable benchmarks for comparing deep learning methods for fluorescence microscopy image denoising.

While dataset diversity is important for training and evaluating fluorescence microscopy denoising methods, few datasets are currently available. One such dataset, introduced by Zhang et al. [11], consists of 12,000 images spanning fluorescence microscopy modalities, including confocal, two-photon, and widefield. This dataset includes samples from cells, zebrafish, and mouse brain tissue, but is limited in both sample diversity and image quality. The W2S dataset introduced by Zhou et al. [12], provides 360 image sets with varying noise levels but is constrained to solely widefield imaging. Weigert et al. [15] introduced a dataset of paired image patches of Planaria to evaluate denoising methods. More recently, Hagen et al. [6] introduced a dataset consisting of 567 paired images ranging from 0.26 to 4.19 megapixels, covering both widefield and confocal imaging modalities. This dataset includes images of actin, mitochondria, nucleus, and membrane samples. However, it remains limited in terms of noise level variation, image sizes, and sample types. A well-suited dataset for training deep learning models should include a diverse range of fluorescence microscopy modalities, objective lenses, noise levels, exposure times, and biological samples.

We introduce a novel collection of 324 full-size, paired images, separated into 17,568 paired image crops, containing a greater diversity of specimens and more extreme noise levels than existing fluorescence microscopy denoising datasets. Previously unrepresented specimens include breast cancer tissue array spots, earthworm, freshwater fish gills, rat testis, rabbit testis, human ovary, and tubulin. Imaging modalities include widefield and spinning disk confocal microscopy. We include more extreme noise levels than in previous datasets by widening the gap between high and low exposure times. This increased diversity of specimens and noise conditions makes our dataset a more comprehensive and well-suited resource for evaluating the performance of fluorescence microscopy denoising models. More extreme noise poses a challenge for denoising methods, since the larger variance in the data makes it more difficult for such methods to determine the underlying clean signal from limited samples. Different specimen types pose a challenge due to the diversity of visual p atterns and varying level of self-similarity in the samples.

In addition to the necessity of large, diverse datasets, deep learning approaches require substantial memory for computation. As a result, denoising of fluorescence microscopy images is limited by GPU memory constraints. To address this challenge, we introduce an image stitching approach that enables large images to be denoised in smaller crops and reassembled [16, 17, 18, 19]. Inspired by panorama stitching techniques [20, 21] we optimize per-tile brightness and contrast terms to compensate for the output variation introduced by learning-based denoising methods, and apply linear blending in the overlap region.

We evaluated the performance of six state-of-the-art deep learning denoising models using our dataset. Our selection included supervised [15, 22], unsupervised [23, 24], and zero-shot [25] methods. BM3D [4] was excluded from our analysis due to its inferior performance compared to CARE [15] in a previous study [6]. Self-Supervised Poisson Gaussian Denoising (SSPG) [23] is an extension of blindspot denoising techniques [26, 27] to support the Poisson-Gaussian noise model which is commonly applied in fluorescence microscopy. Self-Inspired Noise2Noise (SN2N) [24] employs an interpolation technique to generate data for Noise2Noise [28] training. SSPG and SN2N are self-supervised techniques to learn a denoiser from noisy data alone. Noise2Fast, proposed by Lequyer et al. [25], is a zero-shot unsupervised denoising method, meaning that it does not require any training on a separate dataset, and instead learns to denoise directly from a single input image. Noise2Fast relies on a checkerboard-down sampling technique to generate a set of four images from the input noisy image, which are used to train a lightweight feed-forward neural network using Noise2Noise-style self-supervision [28]. Content Aware Image Restoration (CARE), developed by Weigert et al. [15], is a supervised model that utilizes a U-Net architecture [29, 30] to learn mappings from degraded images to denoised versions. Restormer, introduced by Zamir et al. [22], is a supervised denoising model that employs an encoder-decoder Transformer-based architecture. The model's core components include a Multi-Dconv Head Transposed Attention (MDTA) block and a Gated Dconv Feed-Forward Network (GDFN). MDTA aids the model in learning both fine details and broader patterns by combining attention mechanisms and depth-wise convolutions. GDFN improves feature quality by using gates and convolutional layers to refine and enhance each layer's image content. Transformer-based models have outperformed the more commonly-used U-Net in previous studies [31].

Table 1. Overview of the samples and preparations in our dataset.

Dataset(s)	Sample Type	Stain	Sample Source	Catalog Number
1	Actin	Alexa488-pahlloidin	Invitrogen	Fluoroslides #1
2	Breast Cancer Array	H&E Stain	Tissuearray.com	BR249
3	Earthworm	H&E Stain	Eisco	BS18225
4-6	Fish Gill	H&E Stain	Eisco	BS18101
7-8	Rat Testis	H&E Stain	Carolina	316464
9-10	Human Ovary	H&E Stain	Carolina	616024
11	Immature Ovary	H&E Stain	Eisco	BS18222
12	Mitochondria	MitoTracker Red	Invitrogen	Fluoroslides #1
13	Mouse Brain	GFP	Sunjin Lab	-
14	Rabbit Testis	H&E Stain	Amscope	PS50
15	Tubulin	BODIPY FL-GAM	Invitrogen	Fluoroslides #2

Table 2. Overview of dataset imaging conditions. WF indicates widefield and CF indicates spinning disk confocal.

Dataset	Type	No. Images	Sizes [MP]	No. Crops	Exp. Time (Low / High) [ms]	Obj. Mag./NA	Ex. [nm]	Em. [nm]	a	b
1	WF	23	5 - 54.86	1,552	1 / 100	60×/1.42	470	525	0.0092	0.0003
2	WF	1	282	1,024	1 / 100	60×/1.42	530	575	0.0091	-0.0007
3	WF	1	134	400	1 / 600	20×/0.45	530	575	0.0321	-0.0116
4	WF	1	256	576	1 / 100	20×/0.45	530	575	0.0116	-0.0009
5	WF	12	19 - 68	1,328	1 / 300	10×/0.40	530	575	0.0419	-0.0152
6	WF	11	28 -55	1,344	1 / 600	10×/0.40	530	575	0.0385	-0.0148
7	CF	105	4	1,680	20 / 200	60×/1.35	532	575	0.0023	-0.0001
8	CF	100	4	1,600	20 / 500	60 ×/1.35	450	575	0.0146	-0.0032
9	WF	13	28 - 72	1,424	1 / 100	10×/0.4	530	575	0.0171	-0.0028
10	WF	11	28 - 55	1,344	1 / 100	4×/0.16	530	575	0.0177	-0.0025
11	WF	1	90	256	1 / 600	20×/0.45	530	575	0.0356	-0.0123
12	WF	14	5 - 55	1,568	1 / 100	60×/1.42	530	575	0.0197	-0.0048
13	WF	8	36-126	1,216	1 / 100	10×/0.4	470	515	0.0327	-0.0095
14	WF	9	5 - 81	928	1 / 100	10×/0.40	530	575	0.0137	-0.0015
15	WF	14	5 - 55	1,328	1 / 100	60×/1.42	470	515	0.0215	-0.0045

Data Description

We generated fifteen distinct datasets of paired low and high-SNR images composed of specimens from actin, breast cancer array, earthworm, fish gill, rat testes, immature ovaries, human ovaries in active phase, mitochondria, mouse brain, rabbit testes, and tubulin as detailed Table 1. There are 324 images in total, ranging in size from 4.19 to 282.22 megapixels (MP). Imaging conditions varied in light intensity, exposure times, objectives, excitation wavelengths, and stains as detailed in Table 2. We extracted non-overlapping crops of size 512 × 512 pixels from the images, resulting in a total of 17,568 crops, which were then divided into a 90%/10% train/test split for each dataset.

We estimated the noise level of the images using the single-image Poisson-Gaussian noise estimation method of Foi et al. [32]. This approach models the variance of the noisy pixel value z in terms of the clean value x as $\mathrm{Var}[z] = ax + b$, where a and b are the parameters of the model. We estimated the noise parameters for each image crop and report the average values for each dataset in Table 2. Compared to the Fluorescence Microscopy Dataset (FMD) [11], our dataset has similar levels of Poisson noise (a) but generally an order of magnitude higher Gaussian noise (b).

We also estimated the noise level of the images by calculating the PSNR of the raw, low-SNR (noisy) image compared to the high-SNR (clean) image (see Table 3). The average raw PSNR of our datasets ranges from 14.12 to 27.66 dB, with an average of 19.92 dB. For comparison, the raw PSNR in the dataset of Hagen et al. [6] ranged from 18.34 to 29.4 dB, with an average of 24.34 dB, and the FMD dataset [11] had an average raw PSNR of 27.22 dB. Thus our dataset consists of more extreme low-SNR data and tests a previously unexplored boundary of what current denoising methods can handle.

Analyses

Denoising performance. The denoising performance of each method is summarized in Table 3 and visualized in Figure 3. Figures 1 and 2 shows representative low-SNR input images, denoised outputs from each model, and the corresponding high-SNR ground truth images from each dataset, with the “fire” colormap applied to enhance contrast. Restormer consistently achieved the highest average PSNR and SSIM. CARE and Noise2Fast’s comparative performance varied depending on the datasets. The performance of the unsupervised technique SSPG was typically behind the other methods.

Speed. Restormer’s training times ranged from 16-18 hours to several days, while the CARE network trained in approximately 3.5 hours per dataset, and SSPG about 1 hour per dataset. Noise2Fast does not require training on a separate training set. Inference times also varied between models: Restormer required about 0.45 seconds per image, CARE required about 1 second per image, SSPG about 0.20 seconds per image, and Noise2Fast, due to its iterative self-supervised denoising method, between 9 and 40 seconds per image.

Adaptive stitching. The deep learning models introduced variations in light intensity between neighboring denoised crops, as shown in Figure 4. Our adaptive image stitching method reduces these intensity differences across stitched crops compared to naïve stitching. Figure 5 shows an example adaptively stitched Restormer result compared to the corresponding low and high SNR images.

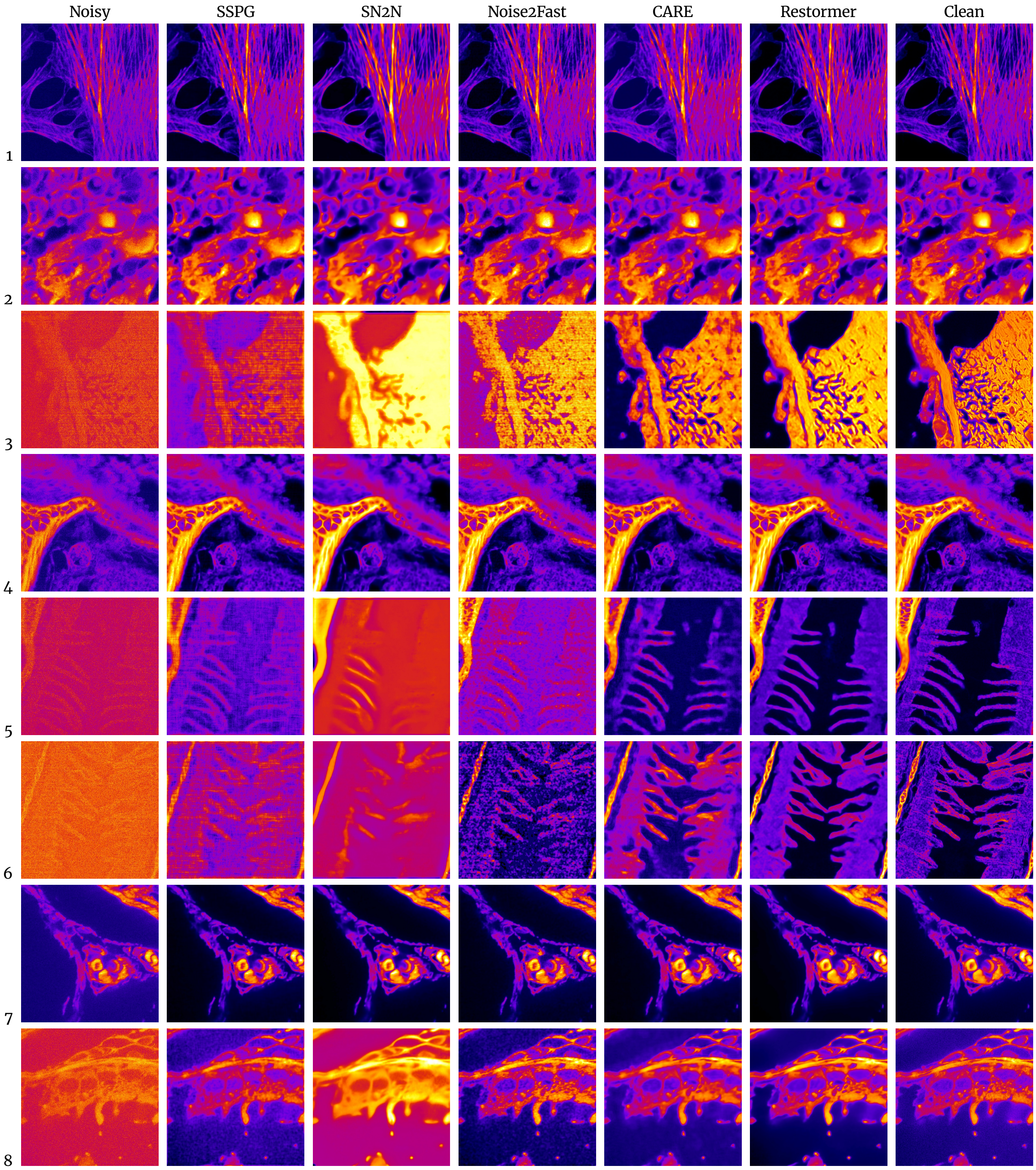

Figure 1. Example noisy input, denoised, and clean images.

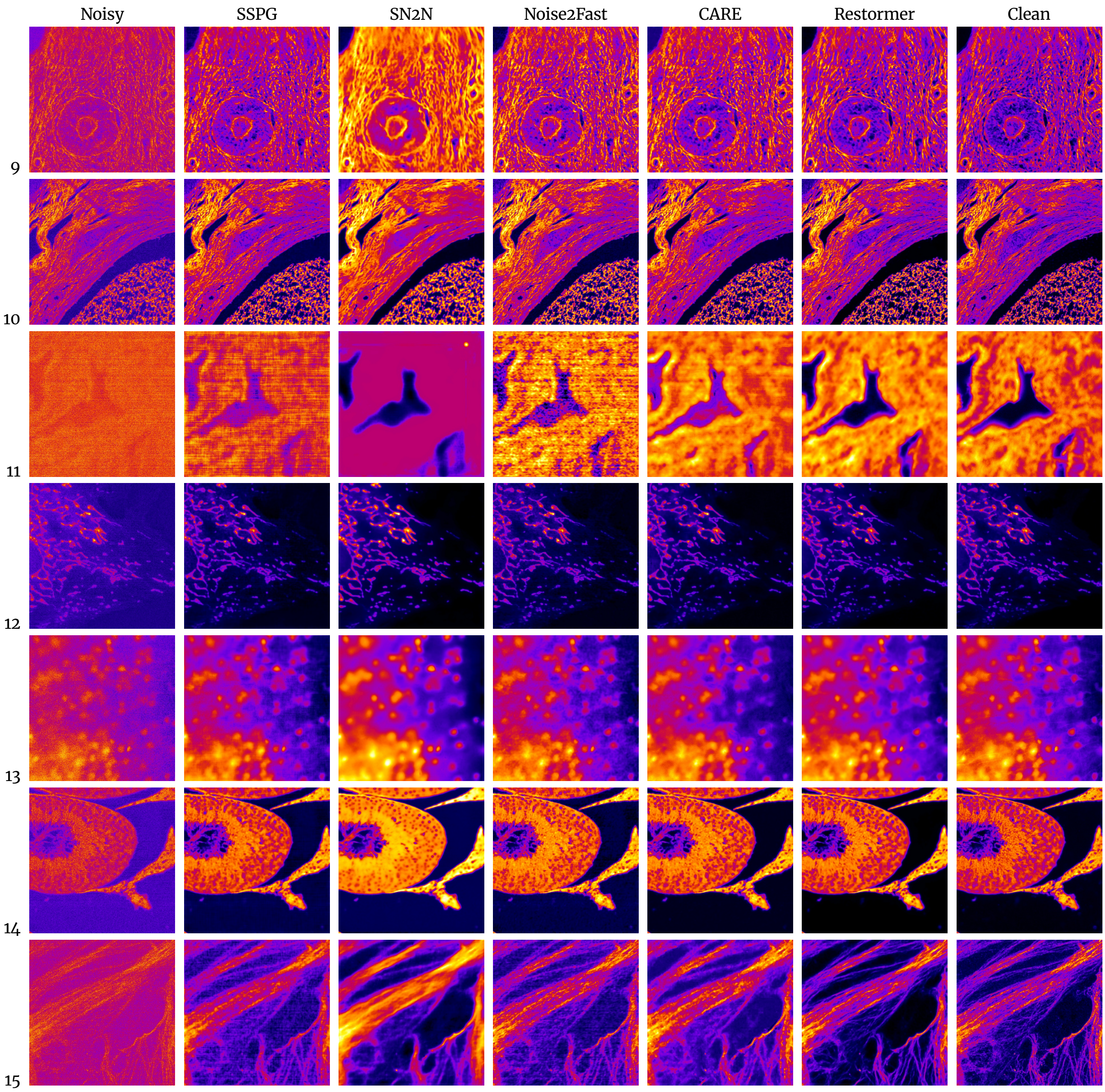

Figure 2. Example noisy input, denoised, and clean images.

Table 3. Average test set results. The highest values per dataset are in bold.

	PSNR						SSIM					
Dataset	Noisy	SSPG	SN2N	Noise2Fast	CARE	Restormer	Noisy	SSPG	SN2N	Noise2Fast	CARE	Restormer
1	23.22	29.05	28.59	29.47	29.50	**31.01**	0.37	0.63	0.63	0.65	0.65	**0.68**
2	23.31	29.83	29.64	30.15	28.07	**34.26**	0.40	0.77	0.77	0.78	0.74	**0.82**
3	17.15	17.68	18.59	18.62	18.54	**19.89**	0.08	0.10	0.13	0.15	0.12	**0.18**
4	22.32	32.17	30.55	32.38	32.04	**33.41**	0.39	0.84	0.80	0.85	0.85	**0.87**
5	15.70	18.51	20.02	20.02	20.83	**23.90**	0.05	0.13	0.29	0.23	0.28	**0.43**
6	27.66	29.51	29.61	29.37	30.28	**33.73**	0.75	0.88	0.88	0.88	0.89	**0.93**
7	18.09	24.52	26.05	26.60	26.60	**27.64**	0.14	0.43	0.47	0.49	0.49	**0.53**
8	15.40	17.80	19.42	19.14	20.33	**23.16**	0.05	0.12	0.27	0.20	0.28	**0.42**
9	19.78	24.80	23.63	25.94	25.88	**26.80**	0.34	0.61	0.49	0.65	0.64	**0.68**
10	21.14	26.25	24.33	26.83	27.03	**28.12**	0.43	0.64	0.55	0.66	0.68	**0.72**
11	14.12	16.21	17.33	17.41	18.06	**21.43**	0.03	0.08	0.22	0.16	0.22	**0.39**
12	22.86	29.42	29.20	30.44	30.38	**32.16**	0.30	0.63	0.63	0.69	0.70	**0.75**
13	18.35	24.01	26.08	25.71	24.43	**28.24**	0.11	0.35	0.46	0.42	0.37	**0.50**
14	21.21	29.07	27.82	29.24	29.35	**30.60**	0.39	0.75	0.71	0.76	0.77	**0.79**
15	18.56	25.99	26.12	27.07	27.15	**28.73**	0.16	0.51	0.53	0.57	0.60	**0.66**

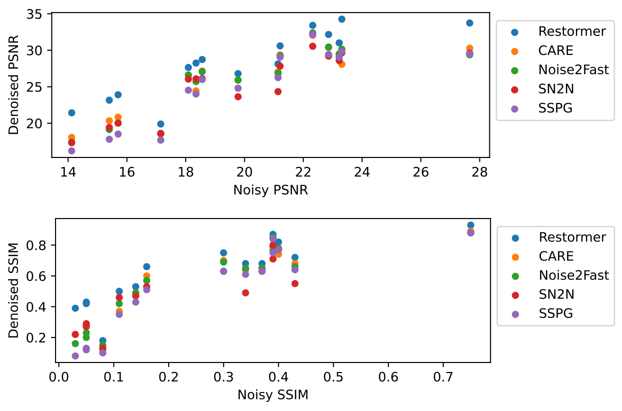

Figure 3. Average noisy vs. denoised PSNR and SSIM for each model and dataset.

Discussion

Across the specimens, imaging modalities, and noise conditions present in our dataset, Restormer achieved the highest performance in terms of PSNR and SSIM. However, due to its supervised training and Transformer-based encoder-decoder architecture, Restormer required substantially longer training times than other supervised models.

We found the denoising performance of SSPG and SN2N to be behind the other methods, which is reasonable considering that the supervised methods (CARE and Restormer) have access to clean data during training, and the zero-shot method (Noise2Fast) trains directly on the test image. Additionally, SSPG, SN2N, and Noise2Fast all make the assumption of independent and identically distributed (i.i.d.) noise, whereas our dataset contains structured noise. Thus the unsupervised methods we tested are likely hindered by the i.i.d. noise assumption.

While its computational costs were considerably higher, Restormer was the most effective model for removing noise while preserving the structural integrity of the original images. Preserving the fine structural detail of fluorescence microscopy images is particularly important because the introduction of artifacts compromises the reliability of downstream biological analysis. This highlights the critical trade-off between computational cost and model accuracy in fluorescence microscopy denoising.

In addition to computational requirements, supervised deep learning models rely on representative, paired image datasets to learn robust mappings from low-SNR to high-SNR images. Denoising performance depends on a model's ability to learn these mappings, but with limited or unrepresentative training datasets, models risk overfitting to specific imaging conditions and fail to handle the inherent variability of live fluorescence microscopy imaging. In contrast, unsupervised approaches do not require extensive training data, but they demonstrated inferior denoising performance, as they struggled to remove noise and maintain the structural integrity of the images in our dataset.

The deep learning models introduced variations in light intensity between the individually denoised neighboring crops, resulting in visible seams and uneven lighting in the reconstructed images. To address the issue of uneven lighting in denoised crops, we implemented an adaptive stitching approach that adjusts each image segment's light intensity based on its neighbors. This method was able to effectively decrease intensity variation across images. Our adaptive stitching process enabled the denoising of large images using deep learning models without exceeding our memory constraints.

Potential implications

Our results highlight the relationship between computational costs and denoising accuracy, underscoring the need for deep learning denoising models that are both computationally efficient and able to remove noise without compromising image integrity. Additionally, given the diversity of biological structures included in our dataset, it would be valuable to explore whether certain deep learning techniques are more effective for denoising specific biological structures. Lastly, since tiny structural distortions can have a significant impact on biological interpretations but may not strongly affect PSNR and SSIM, there is a need for image quality metrics specifically tailored to microscopy images [33].

Methods

Microscopy. We acquired the fluorescence images using an Olympus BX53 microscope equipped with a motorized XY stage (Applied Scientific Imaging, Eugene, OR), Aura III light source (Lumencor, Beaverton, OR), Fluorescence filters (Chroma, Bellows Falls, VT), and Fusion BT camera (Hamamatsu Photonics, Hamamatsu, Japan). The spinning disk confocal setup is described in our previous work [34].

Deep learning methods. Each model was trained and tested using an Nvidia V100 32GB GPU. For each method we used the authors' provided implementations and default settings for training parameters such as loss function, batch size, and learning rate schedule.

For fair comparison, we used consistent data pre-processing and data augmentation procedures across all methods to the extent possible. For pre-processing during training, the images were normalized using percentile normalization. For data augmentation, we horizontally and vertically flipped the images and rotated by 90, 180, and 270 degrees. SSPG, SN2N, CARE and Restormer were trained on patches randomly sampled from the crops. For SSPG, SN2N, and CARE we used 128×128 pixel patches. The recommended approach for Restormer is to increase patch size during training; we began with 128×128 pixel patches and increased to 384×384 pixels by the end of training. Noise2Fast has its own training procedure specific to its efficient and zero-shot design, and so does not use patching or data augmentation.

Image quality metrics. We analyzed the performance of the models on a holdout test set from each dataset not used during training. Performance was quantified using peak signal-to-noise ratio (PSNR) and structural similarity index measure (SSIM) [35] by comparing each denoised result to its corresponding ground truth image. PSNR measures the ratio of the maximum signal power in an image to the power of the noise present in the image. SSIM is based on human visual perception of an image and evaluates image similarity using contrast, luminance, and structure. SSIM values range from zero to one. Higher PSNR and SSIM values indicate increased image similarity and, thus, greater denoising success.

Prior to calculating PSNR and SSIM, images were normalized using the "minimum MSE" normalization method ([15], Supplementary Notes, Section 2.2).

Adaptive image stitching. We prepared the full-size test images for stitching by adding 64-pixel reflective padding around the borders and then dividing the images into 640×640 pixel overlapping crops, so that each crop overlapped with its neighbors by 64 pixels, as illustrated in Figure 6. The central region of each crop, excluding the overlap, represents the valid region of 512×512 pixels.

Crops were denoised using the trained Restormer model, then processed in our adaptive stitching algorithm to re-assemble the crops back into a full-size composite denoised image. We optimized the agreement between overlapping regions by adjusting

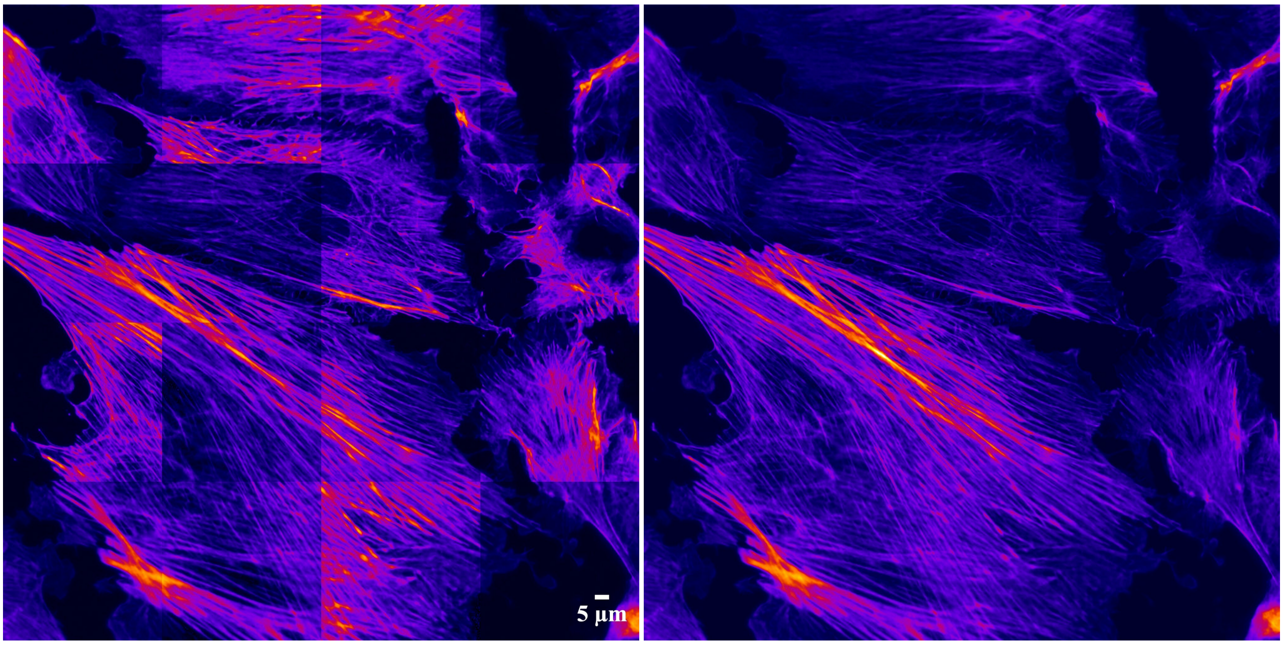

(a) Naïve Stitching **(b)** Adaptive Stitching

Figure 4. Effect of stitching logic on Restormer-denoised images from Dataset 1. (a) Naïve stitching of non-overlapping crops. (b) Adaptive stitching of overlapping crops. Final images are 2048 × 2048 pixels.

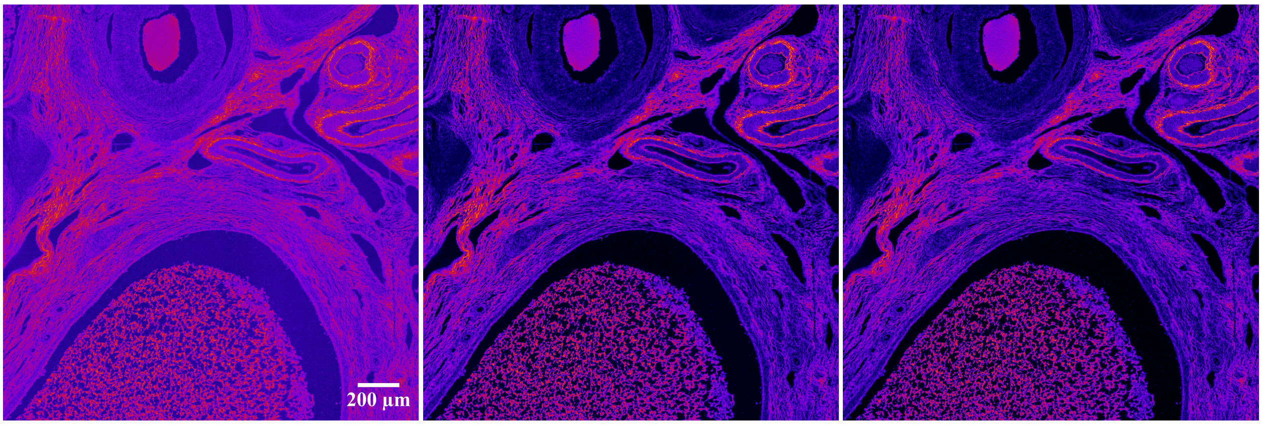

(a) Noisy **(b)** Denoised **(c)** Clean

Figure 5. Example adaptively stitched image from Dataset 9. (a) Noisy image. (b) Adaptive stitching of Restormer denoising results. (c) Clean image. All images are 3072 × 3072 pixels.

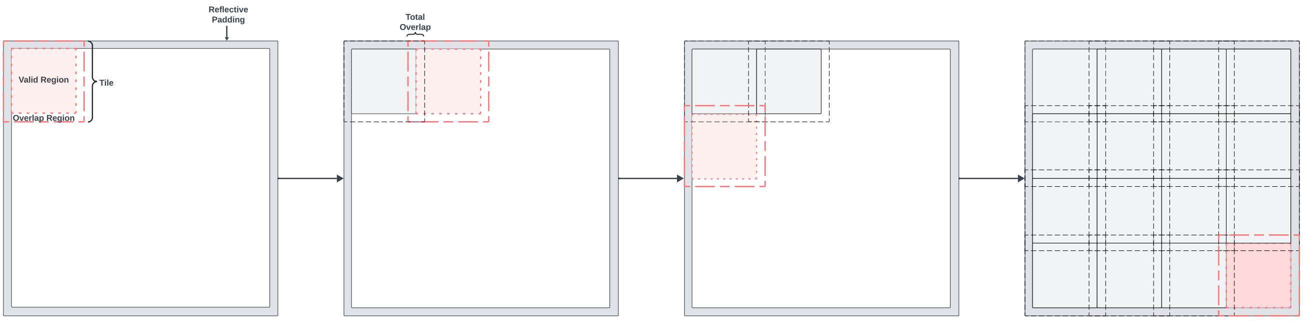

Figure 6. Image cropping logic. The red tile shows the progression of the cropping logic. The shaded area marks the valid region of each tile, while the overlapping region is the space between the valid region and the outer dashed line. Cropping logic proceeds until the entire image is processed.

each crop's scale and shift using the trust region reflective algorithm over a least squares objective. To anchor the adaptive stitching algorithm, we identified the noisy crop with the greatest intensity and assigned its corresponding crop in the denoised image a fixed scale and shift. Following intensity adjustment, a feathered weight mask was applied to each crop, with the weight linearly decreasing from the center of each crop to its edges. In overlapping regions, pixels from all neighboring crops were summed and divided by the total weight at each position, producing smooth transitions between crops.

Availability of source code and requirements

- Project name: Comparison of Deep Learning Approaches for Extreme Low-SNR Image Restoration software and analytics code
- Project home page: https://github.com/nazbuhn/extreme-low-snr-restoration
- Operating systems(s): Platform independent
- Programming language(s): Python

Data availability

All raw and analyzed data is available on GigaDB at http://gigadb.org/site/index. All files and data are distributed under the Creative Commons CC0 waiver, with a request for attribution.

Declarations

List of abbreviations

BM3D: block matching and 3D filtering; CARE: content-aware image restoration; CNN: convolutional neural network; GDFN: Gated-Dcov Feed-Forward Network; GPU: graphics processing unit; MDTA: multi-Dconv transposed attention; MP: megapixels; NA: numerical aperture; PSNR: peak signal-to-noise ratio; ROS: reactive oxygen species; SNR: signal to noise ratio; SSIM: structural similarity index measure; SSPG: Self-Supervised Poisson-Gaussian Denoising

Ethical Approval

Not applicable

Consent for publication

Not applicable

Competing Interests

The authors declare that they have no competing interests.

Funding

This work was supported by the National Institutes of Health grant number 2R15GM128166-02. This work was also supported by the UCCS BioFrontiers Center. The funding sources had no involvement in study design; in the collection, analysis and interpretation of data; in the writing of the report; or in the decision to submit the article for publication.

Author's Contributions

N.E.B: investigation, methodology, software, validation, visualization, writing – original draft, writing – review & editing; S.R.A.: investigation, methodology, software, validation, visualization, writing – original draft; J.H.: data curation; SL: data curation; J.V.: conceptualization, funding acquisition, project administration, software, supervision, validation, writing - review & editing G.H.: conceptualization, data curation, funding acquisition, project administration, resources, supervision, validation, writing – review & editing

Acknowledgements

Not applicable

References

1. Pylvänäinen JW, Gómez-de Mariscal E, Henriques R, Jacquemet G. Live-cell imaging in the deep learning era. Current Opinion in Cell Biology 2023;85:102271.
2. Icha J, Weber M, Waters JC, Norden C. Phototoxicity in live fluorescence microscopy, and how to avoid it. BioEssays 2017;39(8):1700003.
3. Fritzky L, Lagunoff D. Advanced methods in fluorescence microscopy. Analytical Cellular Pathology 2013;36(1-2):5–17.
4. Dabov K, Foi A, Katkovnik V, Egiazarian K. Image denoising by sparse 3-D transform-domain collaborative filtering. IEEE Transactions on Image Processing 2007;16(8):2080–2095.
5. Zhang K, Zuo W, Chen Y, Meng D, Zhang L. Beyond a gaussian denoiser: Residual learning of deep CNN for image denoising. IEEE Transactions on Image Processing 2017;26(7):3142–3155.
6. Hagen GM, Bendesky J, Machado R, Nguyen TA, Kumar T, Ventura J. Fluorescence microscopy datasets for training deep neural networks. GigaScience 2021;10(5):giab032.
7. LeCun Y, Bottou L, Bengio Y, Haffner P. Gradient-based learning applied to document recognition. Proceedings of the IEEE 2002;86(11):2278–2324.
8. Vaswani A, Shazeer N, Parmar N, Uszkoreit J, Jones L, Gomez AN, et al. Attention is all you need. Advances in Neural Information Processing Systems 2017;30.
9. Dosovitskiy A, Beyer L, Kolesnikov A, Weissenborn D, Zhai X, Unterthiner T, et al. An image is worth 16x16 words: Transformers for image recognition at scale. arXiv preprint arXiv:201011929 2020;.
10. Elad M, Kawar B, Vaksman G. Image denoising: The deep learning revolution and beyond—a survey paper. SIAM Journal on Imaging Sciences 2023;16(3):1594–1654.
11. Zhang Y, Zhu Y, Nichols E, Wang Q, Zhang S, Smith C, et al. A Poisson-Gaussian denoising dataset with real fluorescence microscopy images. In: Proceedings of the IEEE/CVF Conference on Computer Vision and Pattern Recognition; 2019. p. 11710–11718.
12. Zhou R, El Helou M, Sage D, Laroche T, Seitz A, Süsstrunk S. W2S: Microscopy data with joint denoising and super-resolution for widefield to SIM mapping. In: European Conference on Computer Vision Springer; 2020. p. 474–491.
13. Qiao C, Li D, Guo Y, Liu C, Jiang T, Dai Q, et al. Evaluation and development of deep neural networks for image super-resolution in optical microscopy. Nature Methods 2021;18(2):194–202.
14. Venkataramanan A, Kloster M, Burfeid-Castellanos A, Dani M, Mayombo NA, Vidakovic D, et al. "UDE DIATOMS in the Wild 2024"': a new image dataset of freshwater diatoms for training deep learning models. GigaScience 2024;13:giae087.
15. Weigert M, Schmidt U, Boothe T, Müller A, Dibrov A, Jain A, et al. Content-aware image restoration: Pushing the limits of

fluorescence microscopy. Nature Methods 2018;15(12):1090–1097.
16. Rankov V, Locke RJ, Edens RJ, Barber PR, Vojnovic B. An algorithm for image stitching and blending. In: Three-dimensional and Multidimensional Microscopy: Image Acquisition and Processing XII, vol. 5701 SPIE; 2005. p. 190–199.
17. Preibisch S, Saalfeld S, Tomancak P. Globally optimal stitching of tiled 3D microscopic image acquisitions. Bioinformatics 2009;25(11):1463–1465.
18. Legesse FB, Chernavskaia O, Heuke S, Bocklitz T, Meyer T, Popp J, et al. Seamless stitching of tile scan microscope images. Journal of Microscopy 2015;258(3):223–232.
19. He B, Zhang Y, Zhang Z, Cheng Y, Zhang F, Sun F, et al. vEMstitch: an algorithm for fully automatic image stitching of volume electron microscopy. GigaScience 2024;13:giae076.
20. Brown M, Lowe DG. Automatic panoramic image stitching using invariant features. International Journal of Computer Vision 2007;74(1):59–73.
21. Ma B, Zimmermann T, Rohde M, Winkelbach S, HeFeng FQ, Lindenmaier W, et al. Use of Autostitch for automatic stitching of microscope images. Micron 2007;38(5):492–499.
22. Zamir SW, Arora A, Khan S, Hayat M, Khan FS, Yang MH. Restormer: Efficient transformer for high-resolution image restoration. In: Proceedings of the IEEE/CVF Conference on Computer Vision and Pattern Recognition; 2022. p. 5728–5739.
23. Khademi W, Rao S, Minnerath C, Hagen G, Ventura J. Self-supervised Poisson-Gaussian denoising. In: Proceedings of the IEEE/CVF Winter Conference on Applications of Computer Vision; 2021. p. 2131–2139.
24. Qu L, Zhao S, Huang Y, Ye X, Wang K, Liu Y, et al. Self-inspired learning for denoising live-cell super-resolution microscopy. Nature Methods 2024;21(10):1895–1908.
25. Lequyer J, Philip R, Sharma A, Hsu WH, Pelletier L. A fast blind zero-shot denoiser. Nature Machine Intelligence 2022;4(11):953–963.
26. Krull A, Buchholz TO, Jug F. Noise2Void - Learning denoising from single noisy images. In: Proceedings of the IEEE/CVF Conference on Computer Vision and Pattern Recognition; 2019. p. 2129–2137.
27. Laine S, Karras T, Lehtinen J, Aila T. High-quality self-supervised deep image denoising. Advances in Neural Information Processing Systems 2019;32.
28. Lehtinen J, Munkberg J, Hasselgren J, Laine S, Karras T, Aittala M, et al. Noise2Noise: Learning Image Restoration without Clean Data. In: International Conference on Machine Learning PMLR; 2018. p. 2965–2974.
29. Ronneberger O, Fischer P, Brox T. U-net: Convolutional networks for biomedical image segmentation. In: International Conference on Medical Image Computing and Computer-Assisted Intervention Springer; 2015. p. 234–241.
30. Falk T, Mai D, Bensch R, Çiçek Ö, Abdulkadir A, Marrakchi Y, et al. U-Net: deep learning for cell counting, detection, and morphometry. Nature Methods 2019;16(1):67–70.
31. Shah ZH, Müller M, Hübner W, Wang TC, Telman D, Huser T, et al. Evaluation of Swin Transformer and knowledge transfer for denoising of super-resolution structured illumination microscopy data. GigaScience 2024;13:giad109.
32. Foi A, Trimeche M, Katkovnik V, Egiazarian K. Practical Poissonian-Gaussian noise modeling and fitting for single-image raw-data. IEEE Transactions on Image Processing 2008;17(10):1737–1754.
33. Gunawan I, Marsh RJ, Aggarwal N, Meijering E, Cox S, Lock JG, et al. Image quality metrics fail to accurately represent biological information in fluorescence microscopy. bioRxiv 2025;p. 2025–08.
34. Hagen GM, Lewis B, Levis S, Hamilton JR, Paul TC. Low-Cost Spinning Disk Confocal Microscopy with a 25-Megapixel Camera. Sensors 2025;25(23):7183.
35. Wang Z, Bovik AC, Sheikh HR, Simoncelli EP. Image quality assessment: from error visibility to structural similarity. IEEE Transactions on Image Processing 2004;13(4):600–612.

Personal Cover
Click here to access/download;Personal Cover;Second Response to Reviewers.pdf

Response to Reviewers

We would like to again thank the reviewers for their careful consideration of our manuscript and thoughtful suggestions for improvement. We have revised our manuscript to address reviewers' comments and provided a version of the revised manuscript with changes marked in blue. A detailed response to each review comment is provided below.

Reviewer #1

1. The authors state that Self-inspired Noise2Noise (SN2N) was tested on two datasets, but "performance was far behind all other methods," and therefore excluded it from the benchmark. However, this conclusion provides no quantitative metrics such as PSNR/SSIM; no training iterations, learning rate, or other key hyperparameter configurations and model convergence information; and no clarification on whether the official implementation or a custom reimplementation was used. N2N methods are unstable and sensitive to hyperparameters. Without the above details, it is impossible to rule out the possibility that performance was underestimated due to insufficient training or improper implementation. It is suggested that the authors clarify the code source and re-evaluate SN2N's performance.

Thank you for these comments. We apologize for the lack of details in our previous response.

We used the authors' implementation of SN2N provided at https://github.com/SR-Wiki/SN2N without modification, following their suggested default settings. To align with the recommendations of SN2N and the preprocessing used for the other methods, we first applied percentile normalization to the images using the default parameters suggested by SN2N. To generate the corresponding patches for N2N training, we used their 2D patch generation script, including eight-fold dataset augmentation with rotations and flips, as used for the other methods tested. We did not apply Patch2Patch data augmentation, following the default settings specified by SN2N and for consistency with the other methods tested. Training was performed according to the defaults specified by SN2N: self-constrained loss weight of 1, batch size of 32, learning rate of 2e-4, and 100 epochs.

We trained and tested SN2N on all our datasets. We updated Table 3 with the SN2N results and Figures 1 and 2 with example images. In general, SN2N was either the worst or second-to-worst performer.

In our discussion section we noted that SSPG, SN2N, and Noise2Fast all make the assumption of independent and identically distributed (i.i.d.) noise, whereas our dataset contains structured noise. Thus the unsupervised methods we tested are likely hindered by the i.i.d. noise assumption.

2. In response to the reviewer's request for characterization of underlying noise statistics, the authors replied using PSNR to estimate noise levels. However, PSNR is an image quality assessment metric that measures the difference between noisy and clean images, rather than the statistical distribution properties of the noise itself (such as Poisson-Gaussian parameters). It is suggested that the authors supplement the variance-mean analysis to estimate Poisson-Gaussian parameters.

Thank you for this suggestion. Following the methods of Zhang et al. (2019) we chose to estimate the Poisson-Gaussian noise model parameters using the single-image estimation method of Foi et al. (2008). We report the average a and b parameter estimates in Table 2 and added a discussion of the results to the Data Description section. Compared to the Fluorescence Microscopy Dataset (Zhang et al., 2019), our dataset has similar levels of Poisson noise (a) but generally an order of magnitude higher Gaussian noise (b).
