# Supplementary material for: Comparison of deep learning approaches for extreme low-SNR image restoration
Source: Gigascience. 2026 Jun 22;15:giag071. doi: 10.1093/gigascience/giag071 (PMC13366542; doi:10.1093/gigascience/giag071)
Supplement: giag071_Authors_Response_To_Reviewer_Comments_original_submission [file giag071_authors_response_to_reviewer_comments_original_submission.pdf]

Author's Response To Reviewer Comments

Response to Reviewers

We thank the reviewers for their careful consideration of our manuscript and thoughtful suggestions for improvement. We have revised our manuscript to address reviewers' comments and provided a version of the revised manuscript with changes marked in blue. A detailed response to each review comment is provided below.

Reviewer #1

"...the specific advantages of this dataset over existing resources (e.g., those cited from Zhang et al., Zhou et al., and Hagen et al.) for advancing low-SNR image restoration are not yet sufficiently clear. In particular, the authors should more explicitly articulate what unique challenges the newly included specimen types and the "extreme-noise" cases introduce, and why these cases provide meaningful validation for assessing model robustness and generalization across imaging conditions."

More extreme noise poses a challenge for denoising methods, since the larger variance in the data makes it more difficult for such methods to determine the underlying clean signal from limited samples. Different specimen types pose a challenge due to the diversity of visual patterns and varying level of self-similarity in the samples.

Please note that in our experiments we do not train general models across datasets and thus do not test model generalization across sample types and imaging conditions. Instead we train and evaluate a separate model instance for each dataset, following common practice in the denoising evaluation literature.

"To substantiate a broader conclusion about unsupervised/self-supervised approaches, the authors are encouraged to broaden the benchmark by adding additional representative unsupervised baselines, such as Noise2Void, Noise2Self, Probabilistic Noise2Void, PPNoise2Void, and Self-inspired Noise2Noise, evaluated under the same training and testing protocol. "

To include a representative unsupervised baseline in benchmark, we evaluated the Self-Supervised Poisson Gaussian Denoising (SSPG) method (Khademi et al., 2021) which is an extension of Noise2Void (Krull et al., 2019; Laine et al., 2019) to support the Poisson-Gaussian noise model which is commonly applied in fluorescence microscopy. In previous experiments (Khademi et al., 2021), SSPG matched or outperformed Fully Unsupervised Probabilistic Noise2Void (PPN2V) (Prakash et al., 2019) on several datasets from FMD (Zhang et al., 2018), and thus we consider it a suitably representative baseline. Note that Probabilistic Noise2Void (PN2V) (Krull et al., 2020) is not truly unsupervised, since it requires access to paired noisy/clean data to fit the noise model.

The results of our expanded experiment are shown in Table 3. We found the performance of SSPG to be behind the other methods, which is reasonable considering that the supervised methods (CARE and Restormer) have access to clean data during training, and the zero-shot method (Noise2Fast) trains directly on the target image.

We also trained and tested Self-inspired Noise2Noise (SN2N) (Qu et al., 2024), another unsupervised method, on two of our datasets; however, the performance was far behind all other methods. Given the long training times required for SN2N, and the uncompetitive performance, we decided to leave SN2N out of the benchmark. However, note that Noise2Fast employs an interpolation scheme similar to SN2N to produce data for Noise2Noise-style training, and thus we feel that the Neighbor2Neighbor (Huang et al., 2021) genre of denoising methods is covered in our benchmark already.

"The performance of the three models is compared under different preprocessing and training pipelines. For a fair and reproducible benchmark, all models should be retrained and evaluated using the same dataset splits and a consistent preprocessing protocol, including data augmentation, normalization schemes, and

cropping/tiling strategies. Otherwise, the observed performance differences may reflect implementation choices rather than the intrinsic capabilities of the models. If certain methods impose specific input constraints (e.g., patch size or channel format), the authors should still minimize such discrepancies as much as possible and clearly justify any unavoidable deviations to ensure the comparison is as equitable as possible. "

To clarify, we used consistent data splits and preprocessing across the methods. All of the original images were cropped into non-overlapping 512x512 pixel crops to form the datasets. We used a consistent 90%/10% train/test split for each dataset and across all methods. Note that Noise2Fast, as a zero-shot method, does not use the train splits. The images were normalized using percentile normalization. For data augmentation, we horizontally and vertically flipped the images and rotated by 90, 180, and 270 degrees. CARE, SSPG and Restormer train on patches randomly sampled from the crops. For CARE and SSPG we used 128x128 pixel patches. The recommended method for Restormer is to increase patch size during training; we began with 128x128 pixel patches and increased to 384x384 pixels by the end of training. Noise2Fast has its own training procedure specific to its efficient and zero-shot design, and so does not use patching or data augmentation.

"The manuscript refers to "extreme low-SNR" conditions, yet it does not characterize the underlying noise statistics (often well described by a Poisson-Gaussian model in fluorescence microscopy). A clear noise characterization is important for interpreting restoration performance and for selecting or designing appropriate denoising algorithms. The authors should estimate the noise statistics from the acquired measurements (e.g., via variance-mean analysis or other established noise-calibration procedures) or justify why such characterization is not feasible in this study. "

We estimate the noise level of the images by calculating the PSNR of the raw, low-SNR (noisy) image compared to the high-SNR (clean) image. We prefer this method of noise characterization because it is model-free and data-driven. The average raw PSNR of our datasets ranges from 14.12 to 27.66 dB, with an average of 19.92 dB. For comparison, the raw PSNR in our previous dataset (Hagen et al., 2021) ranged from 18.34 to 29.4 dB, with an average of 24.34 dB, and the FMD dataset (Zhou et al., 2018) had an average raw PSNR of 27.22 dB. Thus, our dataset consists of more extreme low-SNR data and tests a previously unexplored boundary of what current denoising methods can handle.

Reviewer #2

"I think the way Table 1 is organized is not very clear (at least to me!). Because the table refers to images with different sizes I was quite a bit lost. If for technique A, Sample B, there is an image of 26 MP (which I guess stands for megapixels), is this image then divided in 100 non-overlapping 512x512 images? So of these 100 images 90 are considered for training? How does this really work? In the table, instead of the MP indication, I would put how many paired images are considered for training/testing/validation and their typical size (e.g. technique A, Sample B has 100 images for training, 20 for testing, and 10 for validation. Also I would mention that these images have all size 512x512 or whatever the size was. I actually found a hint of this in the methods section toward the end of the paper. But I would just insert explicit numbers in the table so the reader knows immediately what is happening from the beginning. "

We apologize for the confusion regarding Table 1. Indeed, the original images were cropped into 512x512 pixel non-overlapping patches, which were then divided into a 90%/10% train/test split for each dataset. We added explanatory text to the Data Description section and split the dataset information into Tables 1 and 2 for clarity.

"The term "high-resolution images" is used ambiguously ("We introduce a novel dataset of 324 high-resolution images"). Does this refer to high pixel counts (large field of view), high spatial resolution (sampling frequency/Nyquist), or the optical resolution of the objectives used? A clearer definition is required. "

By “high-resolution” we were referring to the high pixel counts. We have removed the phrase to avoid confusion.

"Given the difference in image sizes across the dataset, some samples appear to contribute disproportionately to the training set (but this point could be due to a misreading on my part of how the table in column 1 and colum 2 is built and how it should be interpreted). Could the authors discuss how this imbalance affects the test results? I would expect under-represented features to show lower performance, and this should be reflected in the evaluation metrics. "

Each sample type / imaging condition forms its own dataset, and we only compute average metrics within each dataset. So, the imbalance in image counts between datasets is not a concern.

"The abstract mentions "spinning disk confocal" as an "also included" category. But before that there is no mention of any other modality. It is critical to define all modalities (e.g., widefield vs. confocal) in the introduction/background, as the difference in Z-resolution and PSF (Point Spread Function) means models trained on one may not generalize to the other. "

We identified which modality was used for each dataset in Table 2 and clarified the corresponding text in the Background section. Note that we train and evaluate a separate model for each dataset, and so we do not test generalization between imaging modalities.

"The authors include excitation wavelengths but omit emission wavelengths. SNR and image quality are in some way dependent on the emission filters and camera quantum efficiency at specific wavelengths; therefore, an emission column should be added to the technical tables. "

We added excitation wavelengths column to Table 2.

"Regarding the layout of Figure 3 I think that for better visual comparison, the figures should be rearranged. Since Restormer is identified as the best-performing model, it should be placed immediately adjacent to the "Ground Truth" (high-SNR) image to allow the reader to easily assess its fidelity. "

We have made the suggested change to Figure 3.

"I'm not sure I got this right but in the abstract the authors mention "12 sub-datasets" but the table contains 15 entries. "

We apologize for the confusion. There are indeed 15 datasets. We clarified the corresponding text.

"I think the sentence " how different stains may impact denoising accuracy" should be rephrased. I can have stains that mark the same structures but with different fluorophores or mechanisms of attachment, and the features will be the same. It is more the target of the stain (which represents the feature content of the image) that would affect how the trained model can be more or less effective on the new target. "

We removed this phrase from the updated manuscript.

em Editorial Manager®

Cookie Preference Center

We use cookies which are necessary to make our site work. We may also use additional cookies to analyze, improve and personalize our content and your digital experience. For more information, see our Cookie Policy.

You may choose not to allow some types of cookies. However, blocking some types may impact your experience of our site and the services we are able to offer. See the different category headings below to find out more or change your settings.

Allow all

Manage Consent Preferences

Strictly Necessary Cookies

Always active

These cookies are necessary for the website to function and cannot be switched off in our systems. They are usually only set in response to actions made by you which amount to a request for services, such as setting your privacy preferences, logging in or filling in forms. You can set your browser to block or alert you about these cookies, but some parts of the site will not then work.

Functional Cookies

These cookies enable the website to provide enhanced functionality and personalisation. They may be set by us or by third party providers whose services we have added to our pages. If you do not allow these cookies then some or all of these services may not function properly.

Performance Cookies

These cookies allow us to count visits and traffic sources so we can measure and improve the performance of our site. They help us to know which pages are the most and least popular and see how visitors move around the site.

Targeting Cookies

These cookies may be set through our site by our advertising partners. They may be used by those companies to build a profile of your interests and show you relevant adverts on other sites. If you do not allow these cookies, you will experience less targeted advertising.

Cookie List

Clear

Apply Cancel
Consent Leg.Interest

Confirm my choices

Power
