# Supplementary material for: Comparison of deep learning approaches for extreme low-SNR image restoration
Source: Gigascience. 2026 Jun 22;15:giag071. doi: 10.1093/gigascience/giag071 (PMC13366542; doi:10.1093/gigascience/giag071)
Supplement: giag071_Authors_Response_To_Reviewer_Comments_revision_1 [file giag071_authors_response_to_reviewer_comments_revision_1.pdf]

# Author's Response To Reviewer Comments

Response to Reviewers

We would like to again thank the reviewers for their careful consideration of our manuscript and thoughtful suggestions for improvement. We have revised our manuscript to address reviewers' comments and provided a version of the revised manuscript with changes marked in blue. A detailed response to each review comment is provided below.

Reviewer #1

"The authors state that Self-inspired Noise2Noise (SN2N) was tested on two datasets, but "performance was far behind all other methods," and therefore excluded it from the benchmark. However, this conclusion provides no quantitative metrics such as PSNR/SSIM; no training iterations, learning rate, or other key hyperparameter configurations and model convergence information; and no clarification on whether the official implementation or a custom reimplementation was used. N2N methods are unstable and sensitive to hyperparameters. Without the above details, it is impossible to rule out the possibility that performance was underestimated due to insufficient training or improper implementation. It is suggested that the authors clarify the code source and re-evaluate SN2N's performance."

Thank you for these comments. We apologize for the lack of details in our previous response.

We used the authors' implementation of SN2N provided at https://github.com/SR-Wiki/SN2N without modification, following their suggested default settings. To align with the recommendations of SN2N and the preprocessing used for the other methods, we first applied percentile normalization to the images using the default parameters suggested by SN2N. To generate the corresponding patches for N2N training, we used their 2D patch generation script, including eight-fold dataset augmentation with rotations and flips, as used for the other methods tested. We did not apply Patch2Patch data augmentation, following the default settings specified by SN2N and for consistency with the other methods tested. Training was performed according to the defaults specified by SN2N: self-constrained loss weight of 1, batch size of 32, learning rate of 2e-4, and 100 epochs.

We trained and tested SN2N on all our datasets. We updated Table 3 with the SN2N results and Figures 1 and 2 with example images. In general, SN2N was either the worst or second-to-worst performer.

In our discussion section we noted that SSPG, SN2N, and Noise2Fast all make the assumption of independent and identically distributed (i.i.d.) noise, whereas our dataset contains structured noise. Thus the unsupervised methods we tested are likely hindered by the i.i.d. noise assumption.

"In response to the reviewer's request for characterization of underlying noise statistics, the authors replied using PSNR to estimate noise levels. However, PSNR is an image quality assessment metric that measures the difference between noisy and clean images, rather than the statistical distribution properties of the noise itself (such as Poisson-Gaussian parameters). It is suggested that the authors supplement the variance-mean analysis to estimate Poisson-Gaussian parameters."

Thank you for this suggestion. Following the methods of Zhang et al. (2019) we chose to estimate the Poisson-Gaussian noise model parameters using the single-image estimation method of Foi et al. (2008). We report the average an and b parameter estimates in Table 2 and added a discussion of the results to the Data Description section. Compared to the Fluorescence Microscopy Dataset (Zhang et al., 2019), our dataset has similar levels of Poisson noise (a) but generally an order of magnitude higher Gaussian noise (b).

## Cookie Preference Center

We use cookies which are necessary to make our site work. We may also use additional cookies to analyze, improve and personalize our content and your digital experience. For more information, see our Cookie Policy.

You may choose not to allow some types of cookies. However, blocking some types may impact your experience of our site and the services we are able to offer. See the different category headings below to find out more or change your settings.

Allow all

## Manage Consent Preferences

### Strictly Necessary Cookies

**Always active**

These cookies are necessary for the website to function and cannot be switched off in our systems. They are usually only set in response to actions made by you which amount to a request for services, such as setting your privacy preferences, logging in or filling in forms. You can set your browser to block or alert you about these cookies, but some parts of the site will not then work.

### Functional Cookies

These cookies enable the website to provide enhanced functionality and personalisation. They may be set by us or by third party providers whose services we have added to our pages. If you do not allow these cookies then some or all of these services may not function properly.

### Performance Cookies

These cookies allow us to count visits and traffic sources so we can measure and improve the performance of our site. They help us to know which pages are the most and least popular and see how visitors move around the site.

**Targeting Cookies**

These cookies may be set through our site by our advertising partners. They may be used by those companies to build a profile of your interests and show you relevant adverts on other sites. If you do not allow these cookies, you will experience less targeted advertising.

**Cookie List**

Clear

Apply Cancel

Consent Leg.Interest

Confirm my choices

Power
