# Supplementary material for: Comparison of deep learning approaches for extreme low-SNR image restoration
Source: Gigascience. 2026 Jun 22;15:giag071. doi: 10.1093/gigascience/giag071 (PMC13366542; doi:10.1093/gigascience/giag071)
Supplement: giag071_Reviewer_1_Report_original_submission — Reviewer 1 -- 1/4/2026 [file giag071_reviewer_1_report_original_submission.pdf]

Reviewer Report

Title: Comparison of Deep Learning Approaches for Extreme Low-SNR Image Restoration

Version: Original Submission **Date:** 1/4/2026

Reviewer name: Reviewer 1

Reviewer Comments to Author:

This manuscript presents a fluorescence microscopy image dataset intended for evaluating deep learning-based image restoration methods across a range of signal-to-noise ratio (SNR) levels. The study benchmarks three models (Restormer, CARE, and Noise2Fast) and introduces an adaptive image-stitching strategy to accommodate large field-of-view images. Overall, the proposed dataset and the reported benchmark results could be valuable to the community. However, the manuscript would benefit from further clarification and strengthening, particularly regarding the depth and rigor of the experimental analysis. Specific comments are as follows:1. The manuscript highlights the novelty and diversity of the proposed dataset, including specimen types that are underrepresented in prior benchmarks as well as examples acquired with spinning-disk confocal microscopy. However, the specific advantages of this dataset over existing resources (e.g., those cited from Zhang et al., Zhou et al., and Hagen et al.) for advancing low-SNR image restoration are not yet sufficiently clear. In particular, the authors should more explicitly articulate what unique challenges the newly included specimen types and the "extreme-noise" cases introduce, and why these cases provide meaningful validation for assessing model robustness and generalization across imaging conditions.2. To substantiate a broader conclusion about unsupervised/self-supervised approaches, the authors are encouraged to broaden the benchmark by adding additional representative unsupervised baselines, such as Noise2Void, Noise2Self, Probabilistic Noise2Void, PPNoise2Void, and Self-inspired Noise2Noise, evaluated under the same training and testing protocol.3. The performance of the three models is compared under different preprocessing and training pipelines. For a fair and reproducible benchmark, all models should be retrained and evaluated using the same dataset splits and a consistent preprocessing protocol, including data augmentation, normalization schemes, and cropping/tiling strategies. Otherwise, the observed performance differences may reflect implementation choices rather than the intrinsic capabilities of the models. If certain methods impose specific input constraints (e.g., patch size or channel format), the authors should still minimize such discrepancies as much as possible and clearly justify any unavoidable deviations to ensure the comparison is as equitable as possible.4. The manuscript refers to "extreme low-SNR" conditions, yet it does not characterize the underlying noise statistics (often well described by a Poisson-Gaussian model in fluorescence microscopy). A clear noise characterization is important for interpreting restoration performance and for selecting or designing appropriate denoising algorithms. The authors should estimate the noise statistics from the acquired measurements (e.g., via variance-mean analysis or other established noise-calibration procedures) or justify why such characterization is not feasible in this study.

Level of Interest

Please indicate how interesting you found the manuscript: Choose an item.

Quality of Written English

Please indicate the quality of language in the manuscript: Choose an item.

Declaration of Competing Interests

Please complete a declaration of competing interests, considering the following questions:

- Have you in the past five years received reimbursements, fees, funding, or salary from an organisation that may in any way gain or lose financially from the publication of this manuscript, either now or in the future?
- Do you hold any stocks or shares in an organisation that may in any way gain or lose financially from the publication of this manuscript, either now or in the future?
- Do you hold or are you currently applying for any patents relating to the content of the manuscript?
- Have you received reimbursements, fees, funding, or salary from an organization that holds or has applied for patents relating to the content of the manuscript?
- Do you have any other financial competing interests?
- Do you have any non-financial competing interests in relation to this paper?

If you can answer no to all of the above, write 'I declare that I have no competing interests' below. If your reply is yes to any, please give details below.

I declare that I have no competing interests.

I agree to the open peer review policy of the journal. I understand that my name will be included on my report to the authors and, if the manuscript is accepted for publication, my named report including any attachments I upload will be posted on the website along with the authors' responses. I agree for my report to be made available under an Open Access Creative Commons CC-BY license (http://creativecommons.org/licenses/by/4.0/). I understand that any comments which I do not wish to be included in my named report can be included as confidential comments to the editors, which will not be published.

Choose an item.

To further support our reviewers, we have joined with Publons, where you can gain additional credit to further highlight your hard work (see: https://publons.com/journal/530/gigascience). On publication of this paper, your review will be automatically added to Publons, you can then choose whether or not to claim your Publons credit. I understand this statement.

Yes Choose an item.
