# Supplementary material for: Comparison of deep learning approaches for extreme low-SNR image restoration
Source: Gigascience. 2026 Jun 22;15:giag071. doi: 10.1093/gigascience/giag071 (PMC13366542; doi:10.1093/gigascience/giag071)
Supplement: giag071_Reviewer_1_Report_revision_1 — Reviewer 1 -- 4/20/2026 [file giag071_reviewer_1_report_revision_1.pdf]

Reviewer Report

Title: Comparison of Deep Learning Approaches for Extreme Low-SNR Image Restoration

Version: Revision 1 **Date:** 4/20/2026

Reviewer name: Reviewer 1

Reviewer Comments to Author:

This manuscript addresses extreme low-SNR fluorescence microscopy image denoising by constructing a new dataset comprising 17,568 paired high- and low-SNR images across 15 sub-datasets with diverse specimen types and imaging conditions. The authors also propose an adaptive image stitching method to address GPU memory constraints when processing large images. The comprehensive benchmark comparing supervised, unsupervised, and zero-shot denoising methods provides insights for the community. However, the following issues still need to be addressed to improve the completeness and reliability:1. The authors state that Self-inspired Noise2Noise (SN2N) was tested on two datasets, but "performance was far behind all other methods," and therefore excluded it from the benchmark. However, this conclusion provides no quantitative metrics such as PSNR/SSIM; no training iterations, learning rate, or other key hyperparameter configurations and model convergence information; and no clarification on whether the official implementation or a custom reimplementation was used. N2N methods are unstable and sensitive to hyperparameters. Without the above details, it is impossible to rule out the possibility that performance was underestimated due to insufficient training or improper implementation. It is suggested that the authors clarify the code source and re-evaluate SN2N's performance.2. In response to the reviewer's request for characterization of underlying noise statistics, the authors replied using PSNR to estimate noise levels. However, PSNR is an image quality assessment metric that measures the difference between noisy and clean images, rather than the statistical distribution properties of the noise itself (such as Poisson-Gaussian parameters). It is suggested that the authors supplement the variance-mean analysis to estimate Poisson-Gaussian parameters.

Level of Interest

Please indicate how interesting you found the manuscript: Choose an item.

Quality of Written English

Please indicate the quality of language in the manuscript: Choose an item.

Declaration of Competing Interests

Please complete a declaration of competing interests, considering the following questions:

- Have you in the past five years received reimbursements, fees, funding, or salary from an organisation that may in any way gain or lose financially from the publication of this manuscript, either now or in the future?
- Do you hold any stocks or shares in an organisation that may in any way gain or lose financially from the publication of this manuscript, either now or in the future?
- Do you hold or are you currently applying for any patents relating to the content of the manuscript?
- Have you received reimbursements, fees, funding, or salary from an organization that holds or has applied for patents relating to the content of the manuscript?
- Do you have any other financial competing interests?
- Do you have any non-financial competing interests in relation to this paper?

If you can answer no to all of the above, write 'I declare that I have no competing interests' below. If your reply is yes to any, please give details below.

NONE.

I agree to the open peer review policy of the journal. I understand that my name will be included on my report to the authors and, if the manuscript is accepted for publication, my named report including any attachments I upload will be posted on the website along with the authors' responses. I agree for my report to be made available under an Open Access Creative Commons CC-BY license (http://creativecommons.org/licenses/by/4.0/). I understand that any comments which I do not wish to be included in my named report can be included as confidential comments to the editors, which will not be published.

I agree to the open peer review policy of the journal

To further support our reviewers, we have joined with Publons, where you can gain additional credit to further highlight your hard work (see: https://publons.com/journal/530/gigascience). On publication of this paper, your review will be automatically added to Publons, you can then choose whether or not to claim your Publons credit. I understand this statement.

Yes Choose an item.
