# Supplementary material for: Comparison of deep learning approaches for extreme low-SNR image restoration
Source: Gigascience. 2026 Jun 22;15:giag071. doi: 10.1093/gigascience/giag071 (PMC13366542; doi:10.1093/gigascience/giag071)
Supplement: giag071_Reviewer_2_Report_original_submission — Reviewer 2 -- 1/13/2026 [file giag071_reviewer_2_report_original_submission.pdf]

Reviewer Report

Title: Comparison of Deep Learning Approaches for Extreme Low-SNR Image Restoration

Version: Original Submission **Date:** 1/13/2026

Reviewer name: Reviewer 2

Reviewer Comments to Author:

In the manuscript, the authors present a dataset of 324 paired high- and low-signal-to-noise ratio fluorescence microscopy images designed to improve the training and benchmarking of deep learning denoising models across various biological specimens and imaging conditions.The authors also developed an image stitching method to address GPU memory constraints when processing large images and demonstrated that supervised transformer-based models achieve the highest denoising performance among state-of-the-art methods.Overall I found the exposition quite good but some parts could result a bit confusing. So while I think the work is useful, I have some comments.Here are my points:I think the way Table 1 is organized is not very clear (at least to me!). Because the table refers to images with different sizes I was quite a bit lost. If for technique A, Sample B, there is an image of 26 MP (which I guess stands for megapixels), is this image then divided in 100 non-overlapping 512x512 images? So of these 100 images 90 are considered for training? How does this really work? In the table, instead of the MP indication, I would put how many paired images are considered for training/testing/validation and their typical size (e.g. technique A, Sample B has 100 images for training, 20 for testing, and 10 for validation. Also I would mention that these images have all size 512x512 or whatever the size was. I actually found a hint of this in the methods section toward the end of the paper. But I would just insert explicit numbers in the table so the reader knows immediately what is happening from the beginning.The term "high-resolution images" is used ambiguously ("We introduce a novel dataset of 324 high-resolution images"). Does this refer to high pixel counts (large field of view), high spatial resolution (sampling frequency/Nyquist), or the optical resolution of the objectives used? A clearer definition is required.Given the difference in image sizes across the dataset, some samples appear to contribute disproportionately to the training set (but this point could be due to a misreading on my part of how the table in column 1 and colum 2 is built and how it should be interpreted). Could the authors discuss how this imbalance affects the test results? I would expect under-represented features to show lower performance, and this should be reflected in the evaluation metrics.The abstract mentions "spinning disk confocal" as an "also included" category. But before that there is no mention of any other modality. It is critical to define all modalities (e.g., widefield vs. confocal) in the introduction/background, as the difference in Z-resolution and PSF (Point Spread Function) means models trained on one may not generalize to the other.The authors include excitation wavelengths but omit emission wavelengths. SNR and image quality are in some way dependent on the emission filters and camera quantum efficiency at specific wavelengths; therefore, an emission column should be added to the technical tables.Regarding the layout of Figure 3 I think that for better visual comparison, the figures should be rearranged. Since Restormer is identified as the best-performing model, it should be placed immediately adjacent to the "Ground Truth" (high-SNR) image to allow the reader to easily assess

its fidelity.I'm not sure I got this right but in the abstract the authors mention "12 sub-datasets" but the table contains 15 entries.I think the sentence " how different stains may impact denoising accuracy" should be rephrased. I can have stains that mark the same structures but with different fluorophores or mechanisms of attachment, and the features will be the same. It is more the target of the stain (which represents the feature content of the image) that would affect how the trained model can be more or less effective on the new target.In conclusion the subject of the paper is interesting in my opinion and I think that overall the authors did a good job in providing very convincing results and in presenting potential applications of their method.The inclusion of a stitching method for high-megapixel images is also a practical contribution for microscopy applications and quite useful.

Level of Interest

Please indicate how interesting you found the manuscript: Choose an item.

Quality of Written English

Please indicate the quality of language in the manuscript: Choose an item.

Declaration of Competing Interests

Please complete a declaration of competing interests, considering the following questions:

- Have you in the past five years received reimbursements, fees, funding, or salary from an organisation that may in any way gain or lose financially from the publication of this manuscript, either now or in the future?
- Do you hold any stocks or shares in an organisation that may in any way gain or lose financially from the publication of this manuscript, either now or in the future?
- Do you hold or are you currently applying for any patents relating to the content of the manuscript?
- Have you received reimbursements, fees, funding, or salary from an organization that holds or has applied for patents relating to the content of the manuscript?
- Do you have any other financial competing interests?
- Do you have any non-financial competing interests in relation to this paper?

If you can answer no to all of the above, write 'I declare that I have no competing interests' below. If your reply is yes to any, please give details below.

I declare that I have no competing interests

I agree to the open peer review policy of the journal. I understand that my name will be included on my report to the authors and, if the manuscript is accepted for publication, my named report including any attachments I upload will be posted on the website along with the authors' responses. I agree for my

report to be made available under an Open Access Creative Commons CC-BY license (http://creativecommons.org/licenses/by/4.0/). I understand that any comments which I do not wish to be included in my named report can be included as confidential comments to the editors, which will not be published.

Choose an item.

To further support our reviewers, we have joined with Publons, where you can gain additional credit to further highlight your hard work (see: https://publons.com/journal/530/gigascience). On publication of this paper, your review will be automatically added to Publons, you can then choose whether or not to claim your Publons credit. I understand this statement.

Yes Choose an item.
